# Operative outcomes of interval cholecystectomy after gallbladder drainage for acute cholecystitis: a systematic review and meta-analysis comparing endoscopic and percutaneous approaches

**DOI:** 10.1186/s12893-026-03644-2

**Published:** 2026-03-09

**Authors:** Hariruk Yodying, Vichit Viriyaroj, Thammanij Rookkachart, Thana Boonsinsukh, Anuwat Chartkitcharoen, Wannakorn Prapasajchavet, Natchanok Mekrugsakit, Patcharaon Petchkaewkul, Ratchanon Laojanun

**Affiliations:** Department of Surgery, HRH Princess Maha Chakri Sirindhorn Medical Center, Faculty of Medicine, Srinakharinwirot University, Nakhon Nayok, Thailand

**Keywords:** Acute cholecystitis, Gallbladder drainage, EUS-guided gallbladder drainage, Endoscopic transpapillary gallbladder drainage, Percutaneous cholecystostomy, Interval cholecystectomy, Conversion to open surgery

## Abstract

**Background:**

Acute cholecystitis in high-risk surgical candidates is frequently managed with gallbladder drainage as a bridge to interval cholecystectomy. Each drainage modality—percutaneous transhepatic (PTGBD), EUS-guided (EUS-GBD), and endoscopic transpapillary (ETGBD)—has distinct anatomical effects that may influence subsequent cholecystectomy. While multiple meta-analyses have established drainage efficacy, comparative evidence regarding operative outcomes of interval surgery remains limited.

**Methods:**

We performed a systematic review and meta-analysis following PRISMA 2020 (PROSPERO: CRD420251232718). Five databases were searched (January 2000–December 2025) for comparative studies reporting operative outcomes of interval cholecystectomy after gallbladder drainage. Primary outcomes were conversion to open cholecystectomy and subtotal cholecystectomy. Random-effects models with Hartung–Knapp adjusted confidence intervals were used. Certainty of evidence was assessed using the GRADE approach.

**Results:**

Ten comparative studies (2019–2025) were included. Meta-analysis of EUS-GBD versus PTGBD (3 studies, *n* = 215) showed no statistically significant difference in conversion to open cholecystectomy (6.4% vs. 16.5%; RR 0.51, 95% CI 0.23–1.13; *P* = 0.07; I²=0%). Meta-analysis of EGBS versus PTGBD (6 studies, *n* = 416) found no statistically significant difference in conversion (18.9% vs. 17.6%; RR 1.14, 95% CI 0.25–5.23; *P* = 0.83; I²=77%) or subtotal cholecystectomy (RR 1.16, 95% CI 0.61–2.18; *P* = 0.52; I²=0%). One RCT comparing ENGBD versus PTGBD (*n* = 22) was synthesized descriptively. Certainty of evidence was low to very low.

**Conclusions:**

No statistically significant differences in operative outcomes of interval cholecystectomy were found between endoscopic and percutaneous gallbladder drainage modalities (low to very low certainty of evidence). Given comparable operative outcomes, drainage modality selection may be guided by drainage efficacy, patient anatomy, and institutional expertise.

**PROSPERO Registration:**

CRD420251232718

**Supplementary Information:**

The online version contains supplementary material available at 10.1186/s12893-026-03644-2.

## Introduction

Acute cholecystitis is a common surgical emergency, and early laparoscopic cholecystectomy is the preferred definitive treatment in suitable candidates [1, 2]. In high-risk patients—particularly the elderly, those with significant comorbidities, or those presenting with severe (Tokyo Grade III) cholecystitis—gallbladder drainage is often performed as a bridge to interval cholecystectomy [3–6].

Percutaneous transhepatic gallbladder drainage (PTGBD) is widely available but creates a transhepatic tract that may result in inflammatory adhesions at the hepatic bed, potentially complicating subsequent surgery. EUS-guided gallbladder drainage (EUS-GBD) using lumen-apposing metal stents provides internal drainage without a transhepatic tract and may improve patient comfort and reduce tube-related complications [7–9]. However, EUS-GBD creates a cholecystoenteric fistula that requires takedown during interval cholecystectomy, raising concerns about operative complexity.

Endoscopic transpapillary gallbladder drainage (ETGBD)—including endoscopic gallbladder stenting (EGBS) and endoscopic naso-gallbladder drainage (ENGBD)—avoids fistula creation but may be technically challenging and could promote cystic duct inflammation or fibrosis, with some authors reporting thickened cystic ducts in up to 60% of patients following EGBS [10]. Multiple meta-analyses and key comparative studies have established that EUS-GBD offers higher technical success and fewer adverse events compared with PTGBD for the drainage phase [11–20]. However, because the ultimate goal of drainage in many patients is definitive cholecystectomy, understanding how different drainage modalities influence subsequent operative outcomes is clinically important.

Key operative endpoints include conversion to open surgery (reflecting operative difficulty and dissection plane complexity), subtotal cholecystectomy as a bailout procedure (indicating inability to achieve critical view of safety [21, 22]), operative time, intraoperative blood loss, and major postoperative complications. Existing comparative studies are limited by small sample sizes, heterogeneous patient selection, and variable reporting of operative denominators (e.g., laparoscopic-intended cases versus all cholecystectomy patients). These factors complicate interpretation and may contribute to substantial heterogeneity.

To our knowledge, no prior systematic review or meta-analysis has specifically synthesized operative outcomes of interval cholecystectomy across gallbladder drainage modalities. Existing syntheses, including the network meta-analysis by Podboy et al. comparing drainage efficacy across EUS-GBD, ETGBD, and PTGBD [14], focus exclusively on drainage-phase endpoints (technical success, clinical success, adverse events) and do not address subsequent surgical outcomes.

We therefore conducted a systematic review and meta-analysis to compare operative outcomes of interval cholecystectomy following EUS-GBD, ETGBD (EGBS/ENGBD), and PTGBD for acute cholecystitis. By addressing this gap, our review aims to complete the clinical picture—integrating surgical outcome data with existing drainage efficacy evidence to enable comprehensive decision-making when selecting drainage modalities in patients planned for interval cholecystectomy.

## Methods

This systematic review was conducted following the Preferred Reporting Items for Systematic Reviews and Meta-Analyses (PRISMA) 2020 guidelines [23] and was prospectively registered with PROSPERO (CRD420251232718). The protocol underwent seven amendments, all documented before examining pooled effect estimates, including changes to population scope, comparison structure, outcome hierarchy, effect measures, and confidence interval methodology.

### Eligibility criteria

We included comparative studies (randomized controlled trials or observational studies) enrolling adults (≥ 18 years) with acute cholecystitis who underwent gallbladder drainage followed by interval laparoscopic cholecystectomy. Eligible interventions were EUS-GBD or ETGBD (EGBS and/or ENGBD), with PTGBD as the comparator. Studies were required to report at least one operative outcome of the interval cholecystectomy. We excluded single-arm studies, case reports, case series with fewer than 10 patients per arm, and studies combining early cholecystectomy patients with those undergoing interval surgery.

### Information sources and search strategy

Five electronic databases were searched from January 1, 2000 to December 2025 (a timeframe selected to encompass the clinical development of endoscopic gallbladder drainage techniques, with EUS-GBD first described in 2007 and EGBS techniques reported from the early 2000s): PubMed/MEDLINE, Embase via Ovid, Scopus, Cochrane Central Register of Controlled Trials (CENTRAL), and ClinicalTrials.gov. The search strategy combined terms for acute cholecystitis, gallbladder drainage modalities, cholecystectomy, and comparative study designs. No language restrictions were applied. Reference lists of included studies and relevant reviews were manually searched, and citation tracking was performed for key articles. The complete search strategies are provided in Supplementary Table S1.

### Study selection

Two reviewers independently screened titles and abstracts using Rayyan systematic review software, followed by full-text assessment of potentially eligible studies. Disagreements were resolved by consensus. Inter-rater reliability was assessed using Cohen’s kappa statistic.

### Data extraction

Data were extracted independently by two reviewers using a standardized form. Variables included study characteristics (author, year, country, design, sample size), population characteristics (age, sex, cholecystitis severity, comorbidity indices), intervention details (drainage technique, stent type, interval to surgery), and all reported operative outcomes.

For conversion to open cholecystectomy, denominators were defined as laparoscopic-intended cases when studies reported planned open cholecystectomy separately (e.g., Katsura 2024). For continuous outcomes (operative time, blood loss), denominators may differ from conversion denominators when studies reported these outcomes for all cholecystectomy patients including those converted to open (e.g., Ishii 2023).

For continuous outcomes reported as median with range, means and standard deviations were estimated using the method of Wan et al. [24]; for median with interquartile range, the method of Luo et al. was applied [25]. Specific conversion formulas are detailed in Supplementary Table S4. When operative outcomes were reported only for the subset of drained patients who proceeded to interval cholecystectomy, we extracted data using the surgical-cohort denominators reported for those outcomes.

### Risk of bias assessment

Risk of bias was assessed using the Cochrane Risk of Bias tool 2.0 (RoB 2) for randomized trials and the Risk Of Bias In Non-randomised Studies of Interventions (ROBINS-I) for observational studies [26, 27]. Assessment was performed independently by two reviewers with disagreements resolved by consensus.

### Outcomes

Primary outcomes were defined as comparison-specific based on clinical relevance and data availability, as pre-specified in the PROSPERO protocol (CRD420251232718). For EUS-GBD versus PTGBD, conversion to open cholecystectomy was the sole primary outcome because none of the three included studies reported subtotal cholecystectomy rates. For EGBS versus PTGBD, two co-primary outcomes were designated—conversion to open cholecystectomy and subtotal cholecystectomy—because subtotal cholecystectomy is a direct measure of operative difficulty at Calot’s triangle, reflecting the anatomical impact of cystic duct stent-induced fibrosis, and the majority of EGBS studies reported this outcome. For ENGBD versus PTGBD, only descriptive synthesis was planned given data limited to a single RCT.

Conversion to open cholecystectomy reflects overall operative difficulty and the inability to safely complete laparoscopic dissection. Subtotal cholecystectomy specifically captures the need for bailout procedures when critical view of safety cannot be achieved, making it particularly relevant for assessing cystic duct fibrosis effects of EGBS. Secondary outcomes included operative time, intraoperative blood loss, and major postoperative complications (Clavien-Dindo grade ≥ III).

### Statistical analysis

Risk ratios (RR) with 95% confidence intervals were calculated for dichotomous outcomes; mean differences (MD) with 95% confidence intervals were calculated for continuous outcomes. Random-effects models using the DerSimonian-Laird τ² estimator were used for all analyses. Given the small number of studies per comparison (k = 2–6), Hartung-Knapp-Sidik-Jonkman (HKSJ) confidence intervals were used as the primary method, which employs a t-distribution with k-1 degrees of freedom to properly account for uncertainty in between-study variance estimation [28, 29]. Statistical heterogeneity was assessed using the Cochran Q test (significance at *P* < 0.10) and I² statistic, interpreted as low (0–40%), moderate (30–60%), substantial (50–90%), or considerable (75–100%) [30].

Studies with zero events in one arm were included using a continuity correction of 0.5; double-zero studies were displayed in forest plots but did not contribute to pooled estimates. Pre-specified sensitivity analyses included fixed-effects versus random-effects models, HKSJ versus conventional Wald-type confidence intervals, exclusion of studies with data quality concerns, and subgroup analysis by study design (propensity score-matched versus unadjusted). Analyses were performed using RevMan Web (Cochrane Collaboration) and R statistical software (meta package).

Network meta-analysis was prospectively planned in the study protocol (PROSPERO CRD420251232718) as a conditional three-node analysis (EUS-GBD vs. EGBS vs. PTGBD) with PTGBD as the common comparator. However, NMA was not feasible because: (1) no studies directly compared EUS-GBD versus EGBS or ENGBD for operative outcomes of interval cholecystectomy, creating a star-shaped network reliant entirely on indirect comparison; (2) the transitivity assumption was materially violated due to systematic differences in patient populations (Western vs. Japanese centers), study designs (propensity-matched vs. unadjusted), and drainage mechanisms between comparisons; and (3) the ENGBD node comprised a single small RCT (*n* = 22). Therefore, pairwise meta-analyses with pre-specified sensitivity analyses were retained as the primary analytical approach, as stipulated in the protocol.

### Certainty of evidence

The certainty of evidence was assessed using the Grading of Recommendations Assessment, Development and Evaluation (GRADE) approach [31]. Summary of Findings tables were generated using GRADEpro GDT.

## Results

### Study selection

**Fig. 1:**
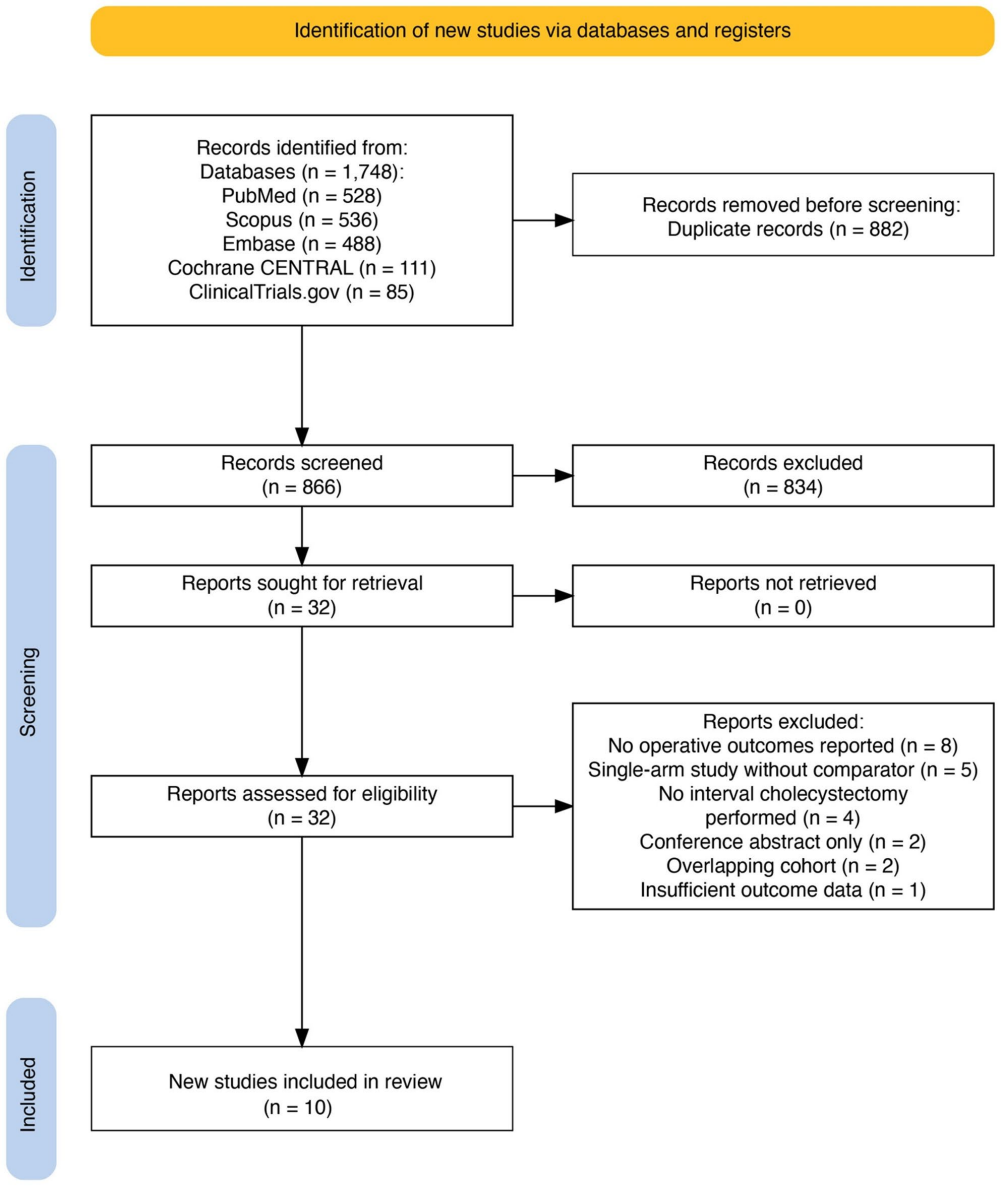
PRISMA 2020 flow diagram for study selection. Records identified from PubMed (*n* = 528), Embase (*n* = 488), Scopus (*n* = 536), Cochrane CENTRAL (*n* = 111), and ClinicalTrials.gov (*n* = 85); total *n* = 1,748. After duplicate removal (*n* = 882), 866 records were screened and 834 excluded. Full-text assessment of 32 articles led to exclusion of 22, with 10 studies meeting inclusion criteria. PRISMA, Preferred Reporting Items for Systematic Reviews and Meta-Analyses

The systematic search identified 1,748 records across five databases. After removing 882 duplicates, 866 records were screened by title and abstract, of which 834 were excluded. Full-text assessment of 32 articles led to exclusion of 22 reports: no operative outcomes reported (*n* = 8), single-arm study without comparator (*n* = 5), no interval cholecystectomy performed (*n* = 4), conference abstract only (*n* = 2), overlapping cohort (*n* = 2), and insufficient outcome data (*n* = 1). Ten studies met all inclusion criteria (Fig. 1).

### Study characteristics

Ten studies published between 2019 and 2025 across four countries (United States, Japan, China, and Australia) were included in this systematic review. Three studies compared EUS-GBD versus PTGBD (*n* = 215 patients undergoing interval cholecystectomy) [32–34]. Six studies compared EGBS versus PTGBD [10, 35–39], with sample sizes varying by outcome due to different denominators for conversion (laparoscopic-intended cases only) versus other outcomes. One randomized controlled trial compared ENGBD versus PTGBD, with operative outcomes reported for the surgical cohort (*n* = 22; 11 per group) [40].

The EUS-GBD studies included the multicenter cohort by Tyberg and colleagues (46 EUS-GBD vs. 92 PTGBD), the single-center series by Ishii and colleagues (35 EUS-GBD vs. 8 PTGBD for laparoscopic-intended cases), and the propensity-matched cohort by Saumoy and colleagues (13 EUS-GBD vs. 21 PTGBD) [32–34]. All employed lumen-apposing metal stents for gallbladder drainage.

The EGBS studies were predominantly from Japan, with one employing propensity score matching (Masuda 2024) and five presenting unadjusted comparisons (Kaura 2020, Kawano 2021, Kaneta 2022, Tanaka 2023, Katsura 2024) [10, 35–39].

The single ENGBD versus PTGBD trial by Mu and colleagues reported operative outcomes for the surgical cohort (*n* = 22; 11 per group) [40]. Detailed study characteristics are presented in Table 1.

**Table 1:** Characteristics of included studies comparing operative outcomes of interval laparoscopic cholecystectomy after gallbladder drainage for acute cholecystitis

Study	Country	Design	Comparison	*N* (I/C)	Age, mean	Male, %	Tokyo III, %	Interval, wk
Tyberg 2023	USA	MC retrospective	EUS-GBD vs. PTGBD	46/92	62.4/65.8	41/47	NR	8.3/9.2
Ishii 2023	Japan	SC retrospective	EUS-GBD vs. PTGBD	35/11ᵇ	71.6/73.9	46/50	23/13	NR
Saumoy 2019	USA	SC retrospective	EUS-GBD vs. PTGBD	13/21	62.5/66.4	31/33	NR	NR
Kaura 2020	Australia	SC retrospective	EGBS vs. PTGBD	52/140	62.9/65.5	58/55	21/29	8.4/10.0
Kawano 2021	Japan	SC retrospective	EGBS vs. PTGBD	18/10	68.3/71.4	61/60	17/20	8.9/9.2
Kaneta 2022	Japan	SC retrospective	EGBS vs. PTGBD	7/26	76/75	57/65	14/19	11.6/9.0
Tanaka 2023	Japan	SC retrospective	EGBS vs. PTGBD	9/34	73.9/73.8	67/71	33/32	6.1/7.1
Katsura 2024	Japan	SC retrospective	EGBS vs. PTGBD	14/45ᶜ	74/73	57/62	43/47	NR
Masuda 2024	Japan	SC PSM	EGBS vs. PTGBD	43/43	68.8/68.4	58/58	33/35	8.9/8.7
Mu 2021	China	RCT	ENGBD vs. PTGBD	11/11ᵃ	71.9/71.9	53/47	27/17	6–8

^a^Surgical cohort only (11 per arm)

^b^For Ishii 2023, PTGBD arm had 11 patients undergoing interval cholecystectomy total; conversion denominator uses laparoscopic-intended cases (*n* = 8) while continuous outcomes use all cholecystectomy patients (*n* = 11)

^c^For Katsura 2024, total enrollment was 14/45; conversion denominator uses laparoscopic-intended cases only (10/37, excluding 4 PTGBD and 0 EGBS planned open cases)

*Abbreviations*: *C* control group, *EGBS *endoscopic gallbladder stenting, *ENGBD *endoscopic naso-gallbladder drainage, *EUS-GBD *endoscopic ultrasound-guided gallbladder drainage, *I *intervention group, *MC *multicenter, *NR *not reported, *PSM *propensity score-matched, *PTGBD *percutaneous transhepatic gallbladder drainage, *RCT *randomized controlled trial, *retrospective *retrospective cohort, *SC *single-center, *Tokyo III *Tokyo Guidelines severity grade III, *wk *weeks

### Risk of bias

Risk of bias assessment using ROBINS-I identified serious concerns in most observational studies, primarily due to confounding by indication (selection bias based on patient severity) and lack of adjustment for prognostic factors. One propensity score-matched study (Masuda 2024) had moderate risk of bias, while all unadjusted studies including Kawano 2021 had serious risk. The single randomized trial (Mu 2021) had some concerns due to open-label design and selective outcome reporting. Domain-level risk of bias assessments are presented in Table 2, with detailed support for judgment provided in Supplementary Table S2.

**Table 2:** Risk of bias assessment for included studies

Study	Tool	D1	D2	D3	D4	D5	D6	D7	Overall
Tyberg 2023	ROBINS-I	Serious	Moderate	Low	Low	Low	Low	Low	Serious
Ishii 2023	ROBINS-I	Serious	Serious	Low	Low	Low	Low	Low	Serious
Saumoy 2019	ROBINS-I	Serious	Moderate	Low	Low	Low	Low	Low	Serious
Kaura 2020	ROBINS-I	Serious	Moderate	Low	Low	Low	Low	Low	Serious
Kawano 2021	ROBINS-I	Serious	Moderate	Low	Low	Low	Low	Low	Serious
Kaneta 2022	ROBINS-I	Serious	Serious	Low	Low	Low	Low	Low	Serious
Tanaka 2023	ROBINS-I	Serious	Moderate	Low	Low	Low	Low	Low	Serious
Katsura 2024	ROBINS-I	Serious	Moderate	Low	Low	Low	Low	Moderate	Serious
Masuda 2024	ROBINS-I	Moderate	Moderate	Low	Low	Low	Low	Low	Moderate
Mu 2021	RoB 2	Some concerns	Some concerns	Low	Low	Some concerns	—	—	Some concerns

*D1–D7* for *ROBINS-I*: *D1* bias due to confounding, *D2 *bias in selection of participants, *D3 *bias in classification of interventions, *D4 *bias due to deviations from intended interventions, *D5 *bias due to missing data, *D6 *bias in measurement of outcomes, *D7 *bias in selection of the reported result

*D1–D5* for *RoB 2*: *D1 *bias arising from the randomization process, *D2 *bias due to deviations from intended interventions, *D3 *bias due to missing outcome data, *D4* bias in measurement of the outcome, *D5 *bias in selection of the reported result

“—” indicates domain not applicable to RoB 2 (5-domain tool)

Support for judgment and detailed rationale are provided in Supplementary Table S2

### Conversion to open cholecystectomy

**Fig. 2:**
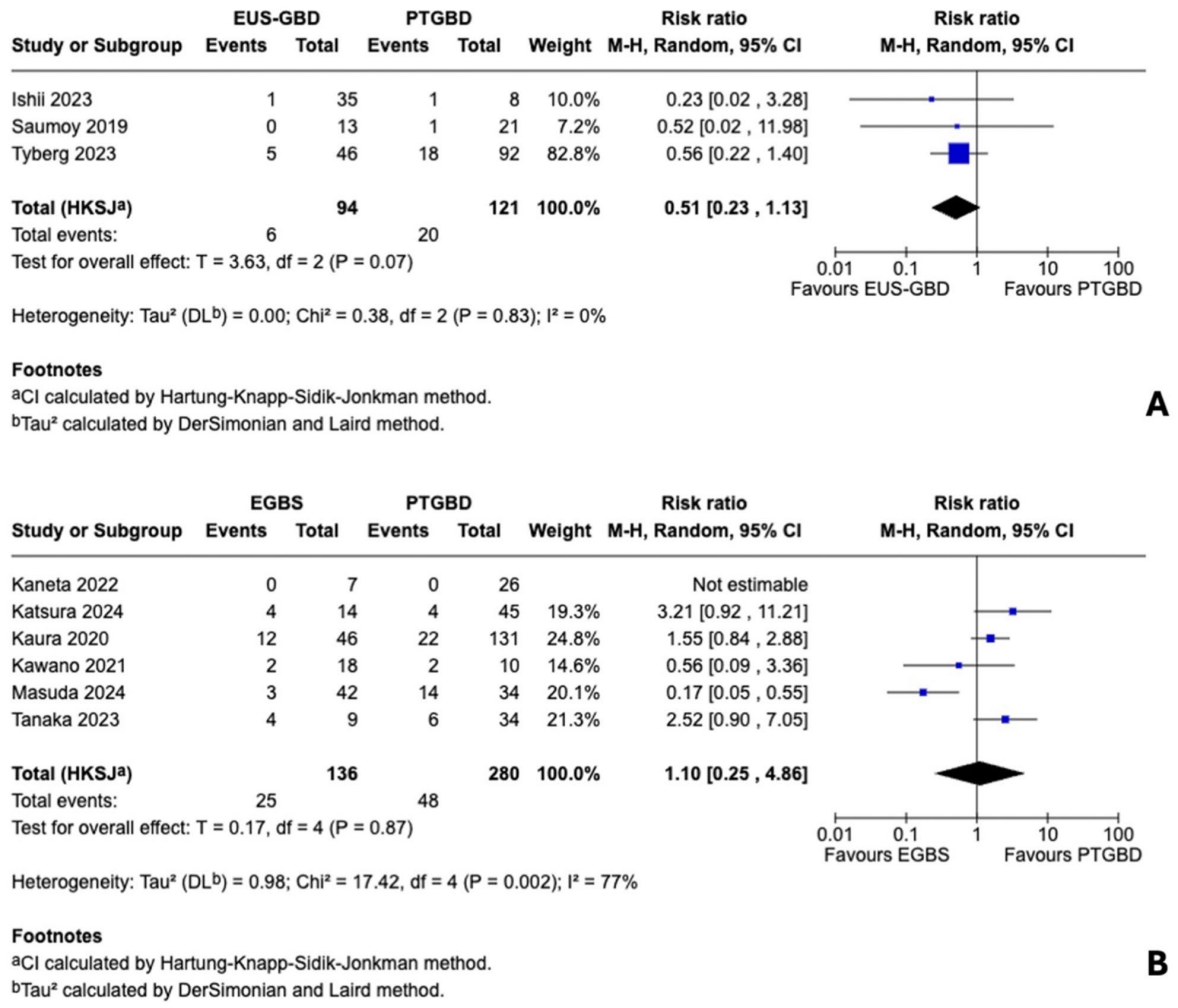
Forest plots for conversion to open cholecystectomy. **A** Endoscopic ultrasound-guided gallbladder drainage (EUS-GBD) versus percutaneous transhepatic gallbladder drainage (PTGBD): pooled risk ratio (RR) 0.51, 95% confidence interval (CI) 0.23–1.13, I²=0%. **B** Endoscopic gallbladder stenting (EGBS) versus PTGBD: pooled RR 1.14, 95% CI 0.25–5.23, I²=77%. Heterogeneity reflects confounding by indication. CI calculated using Hartung-Knapp-Sidik-Jonkman method

For EUS-GBD versus PTGBD, three studies (*n* = 215) reported conversion rates. Conversion occurred in 6 of 94 patients (6.4%) in the EUS-GBD group versus 20 of 121 patients (16.5%) in the PTGBD group. Meta-analysis demonstrated a non-significant 49% relative risk reduction favoring EUS-GBD (RR 0.51, 95% HKSJ CI 0.23–1.13; *P* = 0.07) with absent heterogeneity (I²=0%, Q = 0.38, *P* = 0.83). Notably, all three studies individually favored EUS-GBD, with the multicenter study by Tyberg and colleagues contributing the majority of statistical weight (82.8%). Despite the additional surgical complexity of fistula takedown, the consistent direction across studies suggests that benefits of superior drainage and absence of transhepatic adhesions outweigh the added operative step (Fig. 2A).

For EGBS versus PTGBD, six studies (*n* = 416) reported conversion rates, including one double-zero study (Kaneta 2022: 0/7 vs. 0/26). Conversion occurred in 25 of 132 patients (18.9%) in the EGBS group versus 48 of 272 patients (17.6%) in the PTGBD group. Meta-analysis showed no significant difference (RR 1.14, 95% HKSJ CI 0.25–5.23; *P* = 0.83), but with substantial heterogeneity (I²=77%, Q = 17.23, *P* = 0.002). Exploratory analysis revealed a striking pattern: the single propensity score-matched study (Masuda 2024) strongly favored EGBS (RR 0.17, 95% CI 0.05–0.55), while unadjusted studies showed either neutral or opposite effects. Sensitivity analysis excluding Masuda 2024 yielded a near-homogeneous pooled estimate trending toward higher conversion with EGBS (RR 1.86, 95% HKSJ CI 0.74–4.69; *P* = 0.10; I²=3%), confirming that the observed heterogeneity was driven by this single adjusted study rather than random variation. This discordance suggests confounding by indication—sicker patients selectively undergoing EGBS in unadjusted analyses—rather than true clinical heterogeneity (Fig. 2B; Supplementary Figure S1).

For ENGBD versus PTGBD, the single randomized trial (Mu 2021) reported conversion in 1 of 11 ENGBD patients versus 1 of 11 PTGBD patients (RR 1.00, 95% CI 0.07–14.05), precluding meta-analysis.

### Subtotal cholecystectomy

Subtotal cholecystectomy data were available only for the EGBS comparison, as none of the EUS-GBD studies reported this outcome. Five studies (*n* = 411) reported subtotal cholecystectomy rates, including one double-zero study (Kaura 2020: 0/52 vs. 0/140, reflecting institutional practice). Subtotal cholecystectomy was performed in 13 of 124 patients (10.5%) in the EGBS group versus 34 of 287 patients (11.8%) in the PTGBD group.

**Fig. 3:**
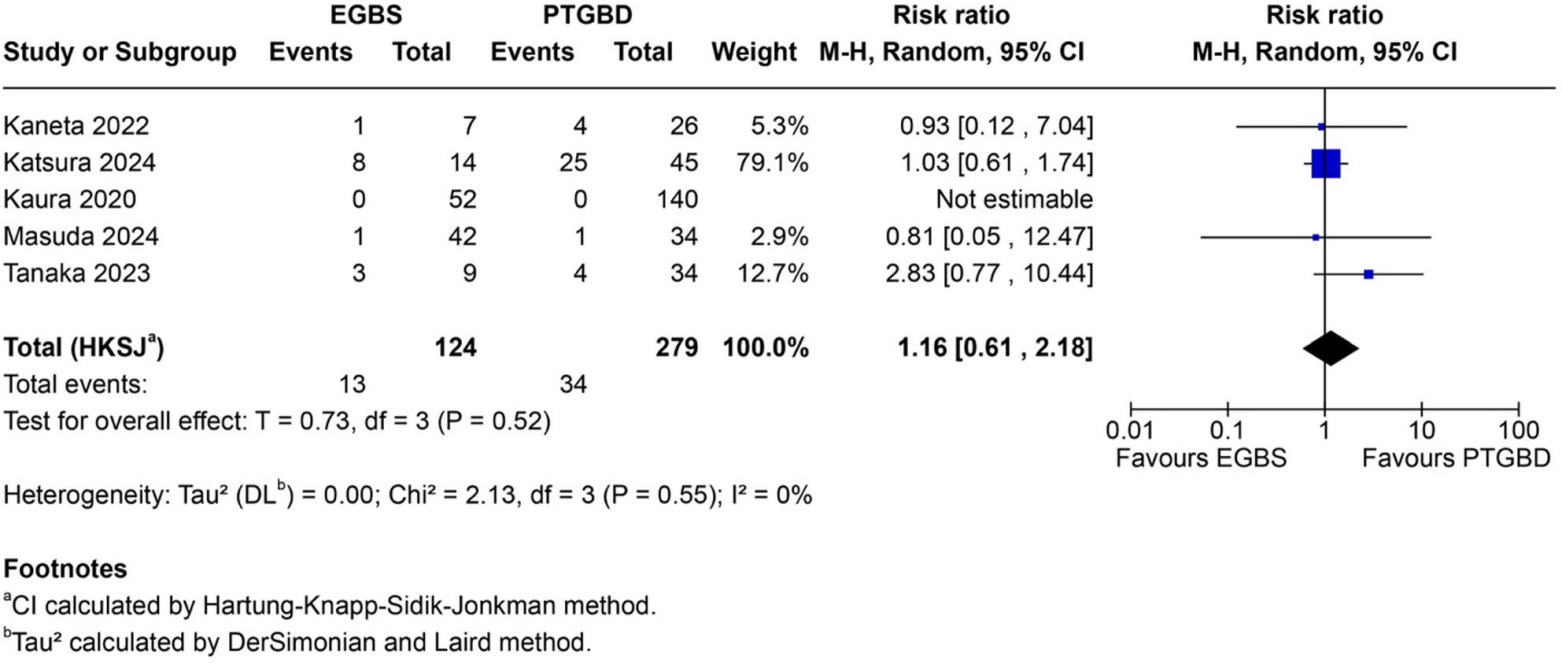
Forest plot for subtotal cholecystectomy comparing EGBS versus PTGBD. Endoscopic gallbladder stenting (EGBS) versus percutaneous transhepatic gallbladder drainage (PTGBD): pooled risk ratio 1.16, 95% confidence interval 0.61–2.18, I²=0%. Homogeneous results indicate similar rates between drainage modalities. CI calculated using Hartung-Knapp-Sidik-Jonkman method

Meta-analysis demonstrated no significant difference (RR 1.16, 95% HKSJ CI 0.61–2.18; *P* = 0.52) with excellent homogeneity (I²=0%, Q = 2.13, *P* = 0.55). This stands in marked contrast to the substantial heterogeneity observed for conversion rates (I²=77%), suggesting subtotal cholecystectomy may represent a more reliable measure of operative difficulty in this context. The Katsura 2024 study contributed the majority of weight (79.1%); sensitivity analysis excluding this study yielded similar conclusions. The homogeneous null finding suggests similar rates between drainage modalities rather than absence of evidence—an “informative null” indicating that EGBS-induced cystic duct fibrosis does not substantially increase the need for bailout procedures (Fig. 3).

### Operative time

**Fig. 4:**
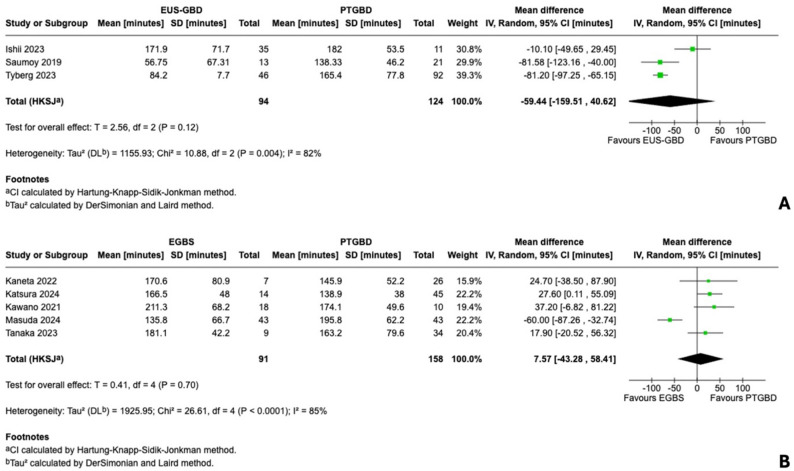
Forest plots for operative time. **A** Endoscopic ultrasound-guided gallbladder drainage (EUS-GBD) versus percutaneous transhepatic gallbladder drainage (PTGBD): three studies (*n* = 218), mean difference − 59.4 min, 95% confidence interval (CI) − 159.5 to 40.6, I²=82%. **B **Endoscopic gallbladder stenting (EGBS) versus PTGBD: five studies (*n* = 249), mean difference + 7.6 min, 95% CI − 43.3 to 58.4, I²=85%

For EUS-GBD versus PTGBD, three studies (*n* = 218) reported operative time. Pooled analysis suggested shorter operative time with EUS-GBD (MD − 59.4 min, 95% CI − 159.5 to 40.6; I²=82%), but with substantial heterogeneity and data quality concerns. The multicenter study by Tyberg and colleagues reported a standard deviation of 7.7 min for EUS-GBD operative time (coefficient of variation 9%), which likely represents a reporting error (standard error reported as standard deviation) given the extreme statistical improbability of such homogeneous operative times across 46 patients at multiple centers. Sensitivity analysis excluding this study reduced the point estimate while maintaining the favorable direction of effect toward EUS-GBD (Fig. 4A; Supplementary Figure S2).

For EGBS versus PTGBD, five studies (*n* = 249) provided operative time data, though four required median-to-mean conversion. Meta-analysis showed no significant difference (MD + 7.6 min, 95% CI − 43.3 to 58.4; I²=85%). The single propensity-matched study with original mean and standard deviation data (Masuda 2024) demonstrated significantly shorter operative time with EGBS (135.8 ± 66.7 vs. 195.8 ± 62.2 min; *P* < 0.001), consistent with the pattern observed for conversion rates (Fig. 4B).

For ENGBD versus PTGBD, the single randomized trial (Mu 2021) reported numerically shorter operative time with ENGBD: median 50 min (IQR 47–90) versus 70 min (IQR 50–104) in the PTGBD group, though this difference was not statistically significant (*P* = 0.25).

### Intraoperative blood loss

**Fig. 5:**
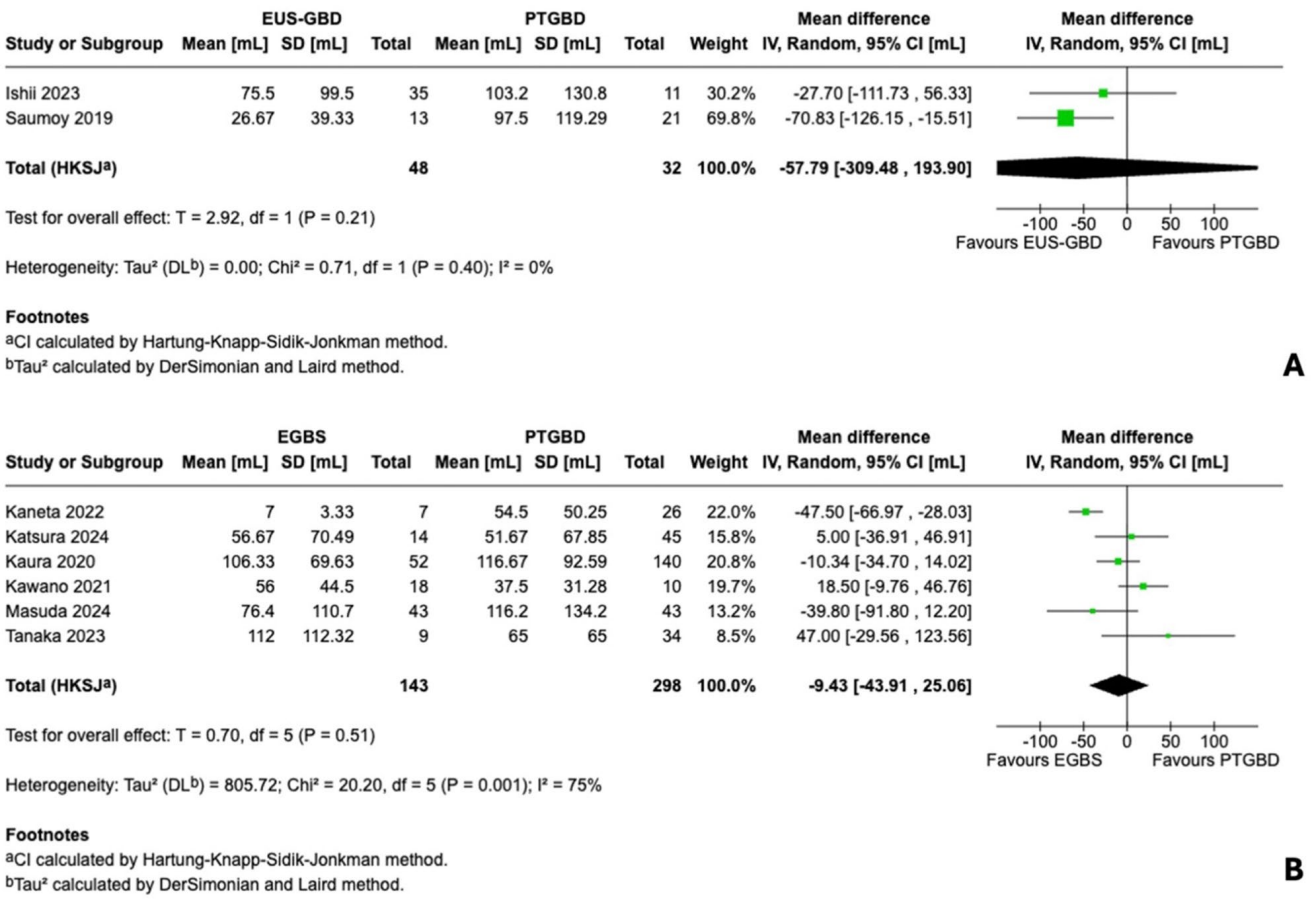
Forest plots for intraoperative blood loss. **A **Endoscopic ultrasound-guided gallbladder drainage (EUS-GBD) versus percutaneous transhepatic gallbladder drainage (PTGBD): two studies (*n* = 80), pooled mean difference − 57.8 mL (95% CI − 309.5 to 193.9, I²=0%), both favoring EUS-GBD (Ishii 2023: 75.5 ± 99.5 vs. 103.2 ± 130.8 mL; Saumoy 2019: 26.7 ± 39.3 vs. 97.5 ± 119.3 mL). Wide confidence interval reflects limited data. **B** Endoscopic gallbladder stenting (EGBS) versus PTGBD: six studies (*n* = 441), pooled mean difference − 9.4 mL (95% CI − 43.9 to 25.1, I²=75%), heterogeneous results with propensity-matched study (Masuda 2024) favoring EGBS

For EUS-GBD versus PTGBD, two studies reported blood loss (*n* = 80; Tyberg 2023 did not report this outcome). Both favored EUS-GBD: Ishii 2023 (75.5 ± 99.5 vs. 103.2 ± 130.8 mL) and Saumoy 2019 (26.67 ± 39.33 vs. 97.50 ± 119.29 mL). Using the pre-specified Hartung–Knapp adjustment, the pooled estimate did not demonstrate a significant difference (MD − 57.8 mL, 95% CI − 309.5 to 193.9; *P* = 0.21; I²=0%). With only two studies (df = 1 for HKSJ), the confidence interval is appropriately wide and results should be interpreted cautiously (Fig. 5A).

For EGBS versus PTGBD, six studies (*n* = 441) reported blood loss, with five requiring median-to-mean conversion. Results were heterogeneous (I²=75%), and pooled analysis did not demonstrate a statistically significant difference (MD − 9.4 mL, 95% CI − 43.9 to 25.1; *P* = 0.51). The propensity-matched Masuda 2024 study demonstrated lower blood loss with EGBS (76.4 ± 110.7 vs. 116.2 ± 134.2 mL) (Fig. 5B).

For ENGBD versus PTGBD, the single randomized trial (Mu 2021) reported significantly lower intraoperative blood loss with ENGBD: median 15 mL (IQR 5–20) versus 40 mL (IQR 20–70) in the PTGBD group (*P* = 0.03). This represented the only statistically significant operative outcome finding in this comparison and was accompanied by lower rates of postoperative abdominal drainage tube placement (27% vs. 82%; *P* = 0.03) and more favorable gallbladder pathology grades (*P* = 0.01), suggesting that ENGBD may result in less tissue trauma and inflammation at the time of cholecystectomy. However, given the small sample size (*n* = 22) and single-center design, these findings should be considered exploratory and hypothesis-generating rather than definitive.

### Major postoperative complications

**Fig. 6:**
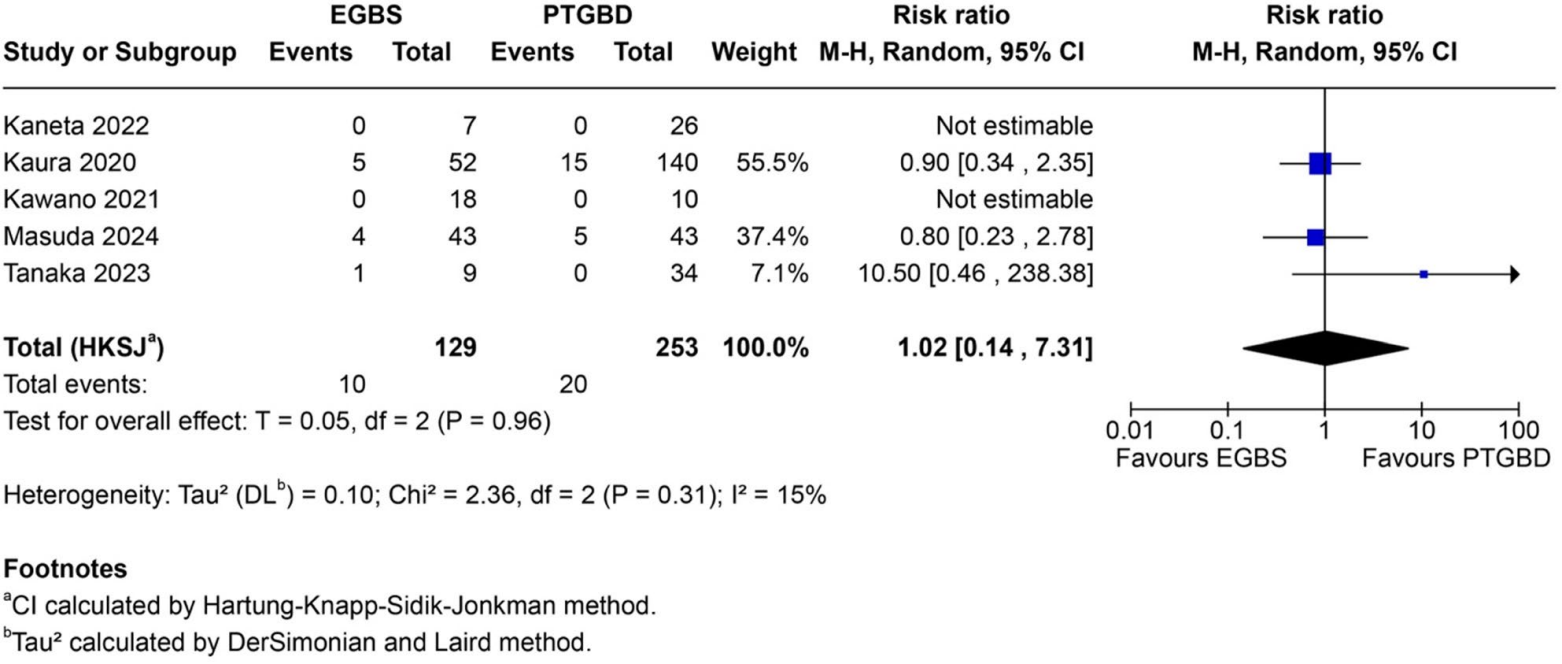
Forest plot for major postoperative complications (Clavien-Dindo ≥ III). Endoscopic gallbladder stenting (EGBS) versus percutaneous transhepatic gallbladder drainage (PTGBD): pooled risk ratio 1.02, 95% confidence interval 0.14–7.31, I²=15%. Two studies with zero events in both groups (Kawano 2021, Kaneta 2022) are displayed but do not contribute to the pooled estimate

Major postoperative complications (Clavien-Dindo ≥ III) could not be meta-analyzed for the EUS-GBD comparison, as none of the three included studies employed Clavien-Dindo classification. For EGBS versus PTGBD, five studies (*n* = 382) reported this outcome. Events occurred in 10 of 129 patients (7.8%) in the EGBS group versus 20 of 253 patients (7.9%) in the PTGBD group. Meta-analysis showed no significant difference (RR 1.02, 95% CI 0.14–7.31; *P* = 0.96; I²=15%), with two double-zero studies (Kawano 2021, Kaneta 2022) (Fig. 6).

### Summary of meta-analysis results

Overall, the meta-analyses did not demonstrate statistically significant differences in operative outcomes between endoscopic and percutaneous drainage modalities. For EUS-GBD versus PTGBD, point estimates consistently favored EUS-GBD across conversion (*P* = 0.07), operative time, and blood loss, but none reached statistical significance; heterogeneity was absent (I²=0%) for the primary outcome. For EGBS versus PTGBD, subtotal cholecystectomy rates were similar (*P* = 0.52; I²=0%), while conversion rates showed substantial heterogeneity (I²=77%) attributable to confounding by study design. The single ENGBD trial (*n* = 22) demonstrated significantly lower blood loss (*P* = 0.03) but no differences in other outcomes. Complete results including effect estimates, confidence intervals, and heterogeneity statistics are presented in Table 3.

**Table 3:** Summary of quantitative findings for operative outcomes of interval cholecystectomy after gallbladder drainage

Comparison	Outcome	k	*n*	Effect	95% CI	*P*	I²
EUS-GBD vs. PTGBD	Conversion to openᵃ	3	215	RR 0.51	0.23–1.13	0.07	0%
	Operative time, minᵇ	3	218	MD − 59.4	−159.5 to 40.6	0.12	82%
	Blood loss, mLᵇ	2	80	MD − 57.8	−309.5 to 193.9	0.21	0%
EGBS vs. PTGBD	Conversion to openᵃ	6	416	RR 1.14	0.25–5.23	0.83	77%
	Subtotal cholecystectomy	5	411	RR 1.16	0.61–2.18	0.52	0%
	Operative time, min	5	249	MD + 7.6	−43.3 to 58.4	0.70	85%
	Blood loss, mL	6	441	MD − 9.4	−43.9 to 25.1	0.51	75%
	Major complicationsᶜ	5	382	RR 1.02	0.14–7.31	0.96	15%
ENGBD vs. PTGBDᵈ	Conversion to open	1	22	RR 1.00	0.07–14.05	—	—
	Operative time, minᵉ	1	22	MD − 12.4	—	0.25	—
	Blood loss, mLᵉ	1	22	Δmedian − 25	—	0.03*	—

^a^Denominator for conversion includes only laparoscopic-intended cases; patients undergoing planned open cholecystectomy were excluded

^b^For Ishii 2023, the denominator for continuous outcomes (*n* = 46) differs from conversion (*n* = 43) because operative time and blood loss were reported for all cholecystectomy patients including those converted to open

^c^Clavien-Dindo grade III or higher; includes two double-zero studies (Kawano 2021, Kaneta 2022) that do not contribute to pooled estimate. ᵈSingle RCT (Mu 2021), surgical cohort only (*n* = 11 per arm); meta-analysis not possible—results are descriptive only. ᵉFor ENGBD, operative time MD calculated from Luo et al. converted means (62.3 vs. 74.7 min). Blood loss reported as median difference (15 vs. 40 mL) with P-value from Mann-Whitney U test. *Statistically significant (*P* < 0.05)

*Abbreviations*: *CI *confidence interval calculated using Hartung-Knapp-Sidik-Jonkman method, *EGBS *endoscopic gallbladder stenting, *ENGBD *endoscopic naso-gallbladder drainage, *EUS-GBD *endoscopic ultrasound-guided gallbladder drainage, *k *number of studies, *MD *mean difference, *n *number of participants, *PTGBD *percutaneous transhepatic gallbladder drainage, *RR *risk ratio

### Certainty of evidence (GRADE assessment)

The certainty of evidence was assessed using the GRADE framework and is presented in Table 4 (Summary of Findings). Overall certainty ranged from very low to low across outcomes.

**Table 4:** GRADE summary of findings for operative outcomes after gallbladder drainage

Outcome	Comparison	k	*n*	Effect [95% CI]	Absolute effectᵃ	Certaintyᵇ
Conversion to open	EUS-GBD vs. PTGBD	3	215	RR 0.51 [0.23–1.13]	81 fewer (127 fewer to 21 more)	⊕⊕◯◯ LOWᶜ˒ᵈ
Conversion to open	EGBS vs. PTGBD	6	416	RR 1.14 [0.25–5.23]	25 more (132 fewer to 745 more)	⊕◯◯◯ VERY LOWᶜ˒ᵈ˒ᵉ
Subtotal cholecystectomy	EGBS vs. PTGBD	5	411	RR 1.16 [0.61–2.18]	19 more (46 fewer to 140 more)	⊕⊕◯◯ LOWᶜ˒ᵈ
Operative time	EUS-GBD vs. PTGBD	3	218	MD − 59.4 min [− 159.5, 40.6]	59 min shorter	⊕◯◯◯ VERY LOWᶜ˒ᵈ˒ᵉ˒ᶠ
Operative time	EGBS vs. PTGBD	5	249	MD + 7.6 min [− 43.3, 58.4]	8 min longer	⊕◯◯◯ VERY LOWᶜ˒ᵈ˒ᵉ˒ᶠ
Blood loss	EUS-GBD vs. PTGBD	2	80	MD − 57.8 mL [− 309.5, 193.9]	58 mL less	⊕◯◯◯ VERY LOWᶜ˒ᵈ˒ᶠ
Blood loss	EGBS vs. PTGBD	6	441	MD − 9.4 mL [− 43.9, 25.1]	9 mL less	⊕◯◯◯ VERY LOWᶜ˒ᵈ˒ᵉ˒ᶠ
Major complicationsᵍ	EGBS vs. PTGBD	5	382	RR 1.02 [0.14–7.31]	2 more (68 fewer to 500 more)	⊕◯◯◯ VERY LOWᶜ˒ᵈ˒ʰ

^a^Per 1000 patients compared with PTGBD baseline risk

^b^GRADE certainty: ⊕⊕⊕⊕ high, ⊕⊕⊕◯ moderate, ⊕⊕◯◯ low, ⊕◯◯◯ very low

^c^Downgraded for risk of bias (observational studies with confounding by indication)

^d^Downgraded for imprecision (wide confidence intervals crossing null, small sample sizes)

^e^Downgraded for inconsistency (I² >50%)

^f^Downgraded for indirectness (median-to-mean data conversion, data quality concerns)

^g^Clavien-Dindo grade III or higher

^h^Downgraded for sparse data (multiple zero-event studies)

*Abbreviations*: *CI *confidence interval, *EGBS *endoscopic gallbladder stenting, *EUS-GBD *endoscopic ultrasound-guided gallbladder drainage, *GRADE *Grading of Recommendations Assessment, Development and Evaluation, *k *number of studies, *MD *mean difference, *n *number of participants, *PTGBD *percutaneous transhepatic gallbladder drainage, *RR *risk ratio

For the EUS-GBD versus PTGBD comparison, the certainty of evidence for conversion to open cholecystectomy was rated low (downgraded one level for risk of bias due to observational designs, and one level for imprecision due to wide confidence intervals crossing the null). Despite consistent results across studies (I²=0%), the small number of events (6 vs. 20) and total sample size (*n* = 215) limited precision.

For the EGBS versus PTGBD comparison, the certainty of evidence for subtotal cholecystectomy was rated low (downgraded for risk of bias and imprecision). Notably, the excellent homogeneity (I²=0%) provided reassurance about consistency of effect. In contrast, conversion to open cholecystectomy was rated very low (downgraded for risk of bias, inconsistency with I²=77%, and imprecision). Sensitivity analysis excluding Masuda 2024 reduced heterogeneity to 3%, confirming that the inconsistency reflects confounding between propensity-matched and unadjusted studies rather than true clinical heterogeneity.

For secondary outcomes (operative time, blood loss, major complications), certainty was rated very low due to additional concerns about indirectness (median-to-mean conversions, variable definitions) and sparse data for complications.

## Discussion

In this systematic review, EUS-GBD was associated with a numerically lower conversion rate to open cholecystectomy compared with PTGBD, with consistent direction of effect across studies (I²=0%) but imprecision due to limited sample size. For EGBS versus PTGBD, pooled estimates across operative endpoints were heterogeneous for conversion but showed similar rates for subtotal cholecystectomy (I²=0%). The certainty of evidence was low to very low due to confounding, imprecision, and heterogeneity.

The clinical pathway for patients with acute cholecystitis unsuitable for early surgery involves two distinct phases—drainage and subsequent surgery—each requiring evidence-based guidance. While existing meta-analyses, including Podboy et al. [14], have addressed drainage efficacy, our findings complete this framework by providing the previously missing surgical outcome data.

For EUS-GBD versus PTGBD, existing meta-analyses establish that EUS-GBD offers superior drainage-phase outcomes: higher technical success (RR 1.04), lower adverse events (RR 0.51), and better quality of life from internal drainage [11–13]. Trial sequential analysis has confirmed these findings achieve statistical credibility with adequate information size [15], and the benefits are particularly pronounced when cautery-enhanced lumen-apposing metal stents are employed, with lower delayed adverse events and shorter hospital stay [12, 41]. A recent systematic review specifically examining interval cholecystectomy after EUS-GBD reported technical success in approximately one-third of patients who proceeded to surgery [42]. Our review adds that surgical outcomes also favor EUS-GBD, with consistent direction of effect favoring lower conversion rates across all included studies. The combined evidence suggests EUS-GBD may be advantageous when endoscopic expertise is available, though certainty remains low and confounding cannot be excluded.

For EGBS versus PTGBD, network meta-analyses suggest similar drainage efficacy between approaches [14]. A nationwide propensity score-matched study from Japan (Ebinuma 2025) provides additional evidence for drainage-phase outcomes, reporting that while EGBS was associated with more frequent long-term postoperative antibiotics therapy (RR 4.7), there were no significant differences in laparotomic cholecystectomy rates between drainage modalities; however, this administrative database study did not report the operative outcomes examined in our review and was therefore excluded from quantitative synthesis [43]. Our finding that surgical outcomes also appear similar—with homogeneous subtotal cholecystectomy rates (I²=0%)—suggests the choice between these modalities should be guided by institutional expertise, technical feasibility, and patient preferences regarding external drainage catheters.

For ENGBD versus PTGBD, evidence is limited to a single randomized trial (Mu 2021, *n* = 22) precluding meta-analysis. This trial provides exploratory and hypothesis-generating findings that require confirmation in larger studies: ENGBD was associated with significantly lower intraoperative blood loss (median 15 vs. 40 mL; *P* = 0.03), lower rates of postoperative abdominal drainage tube placement (27% vs. 82%; *P* = 0.03), and more favorable gallbladder pathology grades (*P* = 0.01) compared with PTGBD. These findings suggest that ENGBD—which provides temporary drainage without inducing cystic duct fibrosis—may preserve more favorable tissue conditions for subsequent cholecystectomy. Unlike EGBS where the indwelling stent remains until surgery, the ENGBD tube is typically removed after bile drainage clears (1–2 weeks), potentially avoiding the inflammatory stimulus that promotes cystic duct fibrosis. However, the small sample size (*n* = 22) and single-center design warrant caution in interpretation, and these findings require validation in larger studies.

Importantly, our non-significant findings for several comparisons should be interpreted as informative evidence rather than “negative” results. When surgical outcomes do not differ significantly between drainage modalities, clinicians can confidently prioritize drainage-phase outcomes without concern for downstream surgical consequences. Specifically, for EGBS versus PTGBD, the homogeneous null result for subtotal cholecystectomy (RR 1.16, I²=0%) answers a clinically important question: cystic duct fibrosis induced by EGBS does not substantially increase bailout procedure rates. This allows the choice between EGBS and PTGBD to be guided by drainage efficacy, patient comfort, and institutional expertise rather than surgical outcome concerns.

Synthesizing drainage and surgical evidence, EUS-GBD emerges as the preferred modality when expertise is available: existing meta-analyses demonstrate fewer drainage-phase adverse events (OR 0.35) and lower reintervention rates (OR 0.18) compared with PTGBD [11, 12, 15], and our data show a consistent direction of effect favoring easier subsequent surgery. For EGBS versus PTGBD, both drainage efficacy and surgical outcomes appear equivalent, making modality selection appropriately driven by technical feasibility and patient factors rather than outcome differences.

The observed surgical outcomes can be understood through the distinct anatomical effects of each drainage modality. EUS-GBD creates a cholecystoenteric fistula requiring surgical takedown, theoretically adding operative complexity. However, our data suggest the net effect remains favorable, likely because internal drainage provides more effective decompression with faster inflammation resolution, and absence of a transhepatic tract eliminates adhesions at the hepatic bed. As hypothesized by Tyberg and colleagues, “because the drainage of the gallbladder obtained during EUS-GBD achieves faster resolution of cholecystitis, dissection of the gallbladder and surrounding structures would be simpler and faster than after PTGBD, even after factoring the reversal of the cholecystogastric or cholecystoduodenal fistula” [32].

For EGBS, the indwelling cystic duct stent induces fibrosis at Calot’s triangle, with Kaneta and colleagues reporting thickened cystic ducts in 60% of EGBS patients versus 0% of PTGBD patients [10]. Theoretical concerns that this fibrosis would increase operative difficulty are not borne out by our findings. The homogeneous null result for subtotal cholecystectomy (I²=0%) suggests that experienced surgeons can manage EGBS-induced fibrosis without substantially increased bailout procedure rates.

Selection bias represents a critical limitation across all comparisons in this review. In non-randomized studies, the choice of drainage modality is inherently influenced by patient severity, anatomical considerations, and institutional expertise—factors that also affect subsequent surgical outcomes. Patients selected for EUS-GBD may represent a different risk profile than those receiving PTGBD, and propensity score matching can only adjust for measured confounders. This fundamental limitation means that observed differences in operative outcomes may reflect patient selection rather than true modality effects.

Several clinical factors likely contribute to the heterogeneity observed across outcomes. First, cholecystitis severity varied across studies (Tokyo Grade II vs. III), and more severe inflammation may independently affect operative difficulty regardless of drainage modality. Second, the interval between drainage and surgery differed substantially (range: 4 weeks to > 6 months), potentially allowing variable degrees of inflammation resolution. Third, surgeon experience and institutional case volume for managing post-drainage cholecystectomy were not reported in any study but likely influence outcomes, particularly for EUS-GBD cases requiring fistula takedown.

The substantial heterogeneity observed for EGBS conversion rates (I²=77%) deserves specific comment. The pattern—a single propensity-matched study (Masuda 2024) strongly favoring EGBS while unadjusted studies favor PTGBD—suggests confounding by indication rather than true clinical heterogeneity. This interpretation is definitively supported by subgroup analysis stratified by study design (Supplementary Figure S1): the unadjusted subgroup, excluding the propensity-matched Masuda 2024, showed markedly reduced heterogeneity (I²=3%) with a homogeneous direction of effect toward higher conversion with EGBS (RR 1.78, 95% HKSJ CI 0.81–3.94; *P* = 0.10). This dramatic heterogeneity reduction confirms that the discordance reflects confounding by indication—sicker or more technically challenging patients may have been preferentially treated with EGBS in unadjusted analyses, leading to worse outcomes attributed to the drainage modality rather than underlying patient factors. The propensity-matched analysis by Masuda and colleagues, which demonstrated substantially lower conversion rates with EGBS (RR 0.17) after adjustment for confounders, likely reflects the true treatment effect when selection bias is controlled.

Based on our two-phase framework integrating drainage and surgical outcomes, we propose the following hierarchy for drainage modality selection, consistent with the principle that “selection of technique will depend on available expertise” [14]. When EUS-GBD expertise is available, this approach should be preferred given superior drainage outcomes plus favorable direction of surgical outcomes; the AGA Clinical Practice Update similarly recommends that EUS-GBD “should be performed by those expert in advanced therapeutic EUS“ [7]. When only ERCP expertise is available, EGBS represents a reasonable alternative, as surgical outcomes appear similar to PTGBD despite theoretical concerns about cystic duct fibrosis. When endoscopic drainage is unavailable, PTGBD remains the reliable standard with well-characterized outcomes, as endorsed by the Tokyo Guidelines 2018 which recommend PTGBD as the first alternative to surgical intervention in surgically high-risk patients [44].

However, for patients who may later become surgical candidates, some data suggest that endoscopic approaches may affect subsequent surgical complexity. Bang and colleagues reported cases where patients who regained surgical candidacy after EUS-GBD required conversion to open surgery or subtotal cholecystectomy due to LAMS-related adhesions [45]. Similarly, Tanaka and colleagues found higher relapse rates during the waiting period and increased operative complexity with EGBS compared with PTGBD [37]. These observations underscore the importance of careful patient selection and coordination with surgeons when choosing drainage modality for patients who may eventually undergo cholecystectomy.

For surgeons performing interval cholecystectomy after drainage, awareness of the drainage modality allows appropriate operative planning. EUS-GBD cases require preparation for fistula takedown, typically with a linear stapler or primary suture closure of the enteric defect. EGBS cases should prompt anticipation of cystic duct fibrosis and potential difficulty with clip application, with readiness to perform subtotal cholecystectomy if Calot’s triangle anatomy cannot be safely defined.

This review has several methodological features worth noting. To our knowledge, no prior meta-analysis has specifically examined surgical outcomes after gallbladder drainage as a bridge to cholecystectomy. The protocol was prospectively registered with amendments documented before data analysis. HKSJ confidence intervals were used given the small number of studies, resulting in wider intervals that reflect the uncertainty inherent in limited evidence. An interpretation framework was pre-specified to guide conclusions regardless of effect direction.

Important limitations must be acknowledged. First, the small number of studies per comparison (k = 2–6) led to wide confidence intervals under HKSJ, limiting precision and precluding formal assessment of publication bias. Second, the predominantly observational designs introduce inherent confounding by indication; only one propensity score-matched study (Masuda 2024) was available for the EGBS comparison [39]. Third, variable definitions of denominators for conversion outcomes (laparoscopic-intended versus all cholecystectomy patients) created challenges for pooled analysis—we used laparoscopic-intended denominators when available and documented discrepancies in footnotes. Fourth, reliance on median-to-mean conversions for several continuous endpoints introduces additional uncertainty; although we used validated methods (Wan/Luo), the converted standard deviations may not accurately reflect the true distributions [24]. Fifth, data quality issues affected several analyses: one study (Tyberg 2023) reported an implausibly low standard deviation requiring sensitivity analysis [32]. Finally, the geographic concentration of EGBS studies (predominantly Japanese) may limit generalizability. Sixth, although network meta-analysis was prospectively planned in our PROSPERO protocol, it could not be conducted due to a disconnected network for operative outcomes, violated transitivity assumptions, and an insufficient ENGBD node (single RCT, *n* = 22); this limits our ability to make indirect comparisons across all three drainage modalities simultaneously. Seventh, Google Scholar was not included as a primary search database because it does not support reproducible Boolean search strategies or controlled vocabulary, limiting its utility for systematic reviews [46]; our five-database strategy exceeds the Cochrane Handbook’s minimum recommendation of CENTRAL, MEDLINE, and Embase [46, 47].

Randomized trials comparing EUS-GBD versus PTGBD with cholecystectomy outcomes as the primary endpoint would provide definitive evidence. Standardized reporting of subtotal cholecystectomy across all drainage studies would strengthen future syntheses. Long-term follow-up studies assessing delayed complications [45, 48, 49], cost-effectiveness analyses incorporating both drainage and surgical phases, timing optimization studies [50–52], and studies examining learning curve effects for EUS-GBD cholecystectomy represent important research priorities.

### Conclusions

This systematic review and meta-analysis did not demonstrate statistically significant differences in operative outcomes of interval cholecystectomy between endoscopic and percutaneous gallbladder drainage modalities. For EUS-GBD versus PTGBD, conversion to open cholecystectomy was not significantly different (low certainty), despite consistent direction of effect across studies. For EGBS versus PTGBD, neither conversion to open cholecystectomy (very low certainty) nor subtotal cholecystectomy (low certainty) differed significantly. A single underpowered RCT comparing ENGBD versus PTGBD showed significantly lower intraoperative blood loss, but this finding is exploratory.

Given comparable operative outcomes, drainage modality selection may be guided by drainage efficacy, patient anatomy, and institutional expertise, with consideration of technical feasibility and patient preferences. Higher-quality comparative studies — ideally multicenter randomized trials — with adequate statistical power and standardized reporting of operative endpoints are needed to determine whether clinically meaningful differences in surgical outcomes exist between drainage modalities.

## Supplementary Information

Supplementary Material 1.

Supplementary Material 2.

## Data Availability

The systematic review protocol was prospectively registered with PROSPERO (CRD420251232718) and is available at [https://www.crd.york.ac.uk/prospero/](https:/www.crd.york.ac.uk/prospero) . Extracted datasets generated during this review, including study-level data and meta-analysis outputs, are available from the corresponding author upon reasonable request. Complete search strategies, risk of bias assessments, detailed statistical analyses, and additional results are provided in the Supplementary Materials accompanying this manuscript. The statistical code used for meta-analyses is available in the study protocol.

## Abbreviations

CI: Confidence interval
EGBS: Endoscopic gallbladder stenting
ENGBD: Endoscopic naso-gallbladder drainage
EUS-GBD: Endoscopic ultrasound-guided gallbladder drainage
GRADE: Grading of recommendations assessment, development and evaluation
HKSJ: Hartung-Knapp-Sidik-Jonkman
LC: Laparoscopic cholecystectomy
MD: Mean difference
NOS: Newcastle-Ottawa scale
PRISMA: Preferred reporting items for systematic reviews and meta-analyses
PROSPERO: International prospective register of systematic reviews
PTGBD: Percutaneous transhepatic gallbladder drainage
RCT: Randomized controlled trial
ROBINS-I: Risk of bias in non-randomized studies of interventions
RR: Risk ratio
TSA: Trial sequential analysis
