# Supplementary material for: Operative outcomes of interval cholecystectomy after gallbladder drainage for acute cholecystitis: a systematic review and meta-analysis comparing endoscopic and percutaneous approaches
Source: BMC Surg. 2026 Mar 9;26:268. doi: 10.1186/s12893-026-03644-2 (PMC13085629; doi:10.1186/s12893-026-03644-2)
Supplement: Supplementary file 1 — Supplementary Material 1. [file 12893_2026_3644_MOESM1_ESM.docx]

**PRISMA 2020 Checklist**

**Title:** Operative Outcomes of Interval Cholecystectomy After Gallbladder Drainage for Acute Cholecystitis: A Systematic Review and Meta-Analysis Comparing Endoscopic and Percutaneous Approaches

| **Section and Topic** | **Item #** | **Checklist Item** | **Location** |
| --- | --- | --- | --- |
| **TITLE** |  |  |  |
| Title | 1 | Identify the report as a systematic review. | Title page, p.1 — "A Systematic Review and Meta-Analysis" |
| **ABSTRACT** |  |  |  |
| Abstract | 2 | See the PRISMA 2020 for Abstracts checklist. | Abstract, p.1 — Structured abstract with Background, Methods, Results, Conclusions |
| **INTRODUCTION** |  |  |  |
| Rationale | 3 | Describe the rationale for the review in the context of existing knowledge. | Introduction, p.2–3 — Discusses existing meta-analyses on drainage efficacy, identifies gap in surgical outcome evidence |
| Objectives | 4 | Provide an explicit statement of the objective(s) or question(s) the review addresses. | Introduction, p.3 — "We therefore conducted a systematic review and meta-analysis to compare operative outcomes of interval cholecystectomy following EUS-GBD, ETGBD (EGBS/ENGBD), and PTGBD for acute cholecystitis." |
| **METHODS** |  |  |  |
| Eligibility criteria | 5 | Specify the inclusion and exclusion criteria for the review and how studies were grouped for the syntheses. | Methods, Eligibility Criteria section, p.3 — Inclusion: comparative studies, adults ≥18 years, acute cholecystitis, gallbladder drainage followed by interval LC. Exclusion: single-arm studies, case reports, <10 patients per arm |
| Information sources | 6 | Specify all databases, registers, websites, organisations, reference lists and other sources searched or consulted to identify studies. Specify the date when each source was last searched or consulted. | Methods, Information Sources section, p.3–4 — PubMed/MEDLINE, Embase via Ovid, Scopus, Cochrane CENTRAL, ClinicalTrials.gov; searched January 2000 to December 2025; reference lists and citation tracking also performed |
| Search strategy | 7 | Present the full search strategies for all databases, registers and websites, including any filters and limits used. | Supplementary Table S1 — Complete search strategies for all 5 databases with line-by-line search terms and results |
| Selection process | 8 | Specify the methods used to decide whether a study met the inclusion criteria of the review, including how many reviewers screened each record and each report retrieved, whether they worked independently, and if applicable, details of automation tools used in the process. | Methods, Study Selection section, p.4 — Two reviewers independently screened using Rayyan software; disagreements resolved by consensus; Cohen's kappa for inter-rater reliability |
| Data collection process | 9 | Specify the methods used to collect data from reports, including how many reviewers collected data from each report, whether they worked independently, any processes for obtaining or confirming data from study investigators, and if applicable, details of automation tools used in the process. | Methods, Data Extraction section, p.4 — Data extracted independently by two reviewers using standardized form; discrepancies resolved by consensus |
| Data items | 10a | List and define all outcomes for which data were sought. Specify whether all results that were compatible with each outcome domain in each study were sought (e.g. for all measures, time points, analyses), and if not, the methods used to decide which results to collect. | Methods, Outcomes section, p.4 — Primary: conversion to open cholecystectomy (EUS-GBD), conversion + subtotal cholecystectomy (EGBS). Secondary: operative time, blood loss, major complications (Clavien-Dindo ≥III) |
|  | 10b | List and define all other variables for which data were sought (e.g. participant and intervention characteristics, funding sources). Describe any assumptions made about any missing or unclear information. | Methods, Data Extraction section, p.4 — Study characteristics, population characteristics (age, sex, severity, comorbidity), intervention details (drainage technique, stent type, interval to surgery) |
| Study risk of bias assessment | 11 | Specify the methods used to assess risk of bias in the included studies, including details of the tool(s) used, how many reviewers assessed each study and whether they worked independently, and if applicable, details of automation tools used in the process. | Methods, Risk of Bias Assessment section, p.4 — Cochrane RoB 2 for RCTs, ROBINS-I for observational studies; assessed independently by two reviewers; disagreements resolved by consensus |
| Effect measures | 12 | Specify for each outcome the effect measure(s) (e.g. risk ratio, mean difference) used in the synthesis or presentation of results. | Methods, Statistical Analysis section, p.4–5 — Risk ratios (RR) for dichotomous outcomes; mean differences (MD) for continuous outcomes |
| Synthesis methods | 13a | Describe the processes used to decide which studies were eligible for each synthesis (e.g. tabulating the study intervention characteristics and comparing against the planned groups for each synthesis (item #5)). | Methods, Statistical Analysis section, p.4–5 — Studies grouped by comparison (EUS-GBD vs PTGBD, EGBS vs PTGBD, ENGBD vs PTGBD); comparison-specific primary outcomes based on data availability |
|  | 13b | Describe any methods required to prepare the data for presentation or synthesis, such as handling of missing summary statistics, or data conversions. | Methods, Data Extraction section, p.4 + Supplementary Table S4 — Median-to-mean conversion using Wan et al. (median with range) and Luo et al. (median with IQR) methods |
|  | 13c | Describe any methods used to tabulate or visually display results of individual studies and syntheses. | Methods, p.4–5 — Forest plots generated using RevMan Web; results presented in Table 2 |
|  | 13d | Describe any methods used to synthesize results and provide a rationale for the choice(s). If meta-analysis was performed, describe the model(s), method(s) to identify the presence and extent of statistical heterogeneity, and software package(s) used. | Methods, Statistical Analysis section, p.4–5 — Random-effects models (DerSimonian-Laird τ²); HKSJ confidence intervals for small number of studies; heterogeneity assessed by Cochran Q test and I² statistic; RevMan Web and R (meta package) |
|  | 13e | Describe any methods used to explore possible causes of heterogeneity among study results (e.g. subgroup analysis, meta-regression). | Methods, Statistical Analysis section, p.5 — Subgroup analysis by study design (propensity score-matched vs unadjusted) |
|  | 13f | Describe any sensitivity analyses conducted to assess robustness of the synthesized results. | Methods, Statistical Analysis section, p.5 — Fixed vs random effects, HKSJ vs Wald-type CI, exclusion of studies with data quality concerns, subgroup by study design |
| Reporting bias assessment | 14 | Describe any methods used to assess risk of bias due to missing results in a synthesis (arising from reporting biases). | Methods, p.5 + Discussion Limitations, p.8 — Funnel plots not feasible due to <10 studies per comparison; acknowledged as limitation |
| Certainty assessment | 15 | Describe any methods used to assess certainty (or confidence) in the body of evidence for an outcome. | Methods, Certainty of Evidence section, p.5 — GRADE approach; Summary of Findings tables generated using GRADEpro GDT |
| **RESULTS** |  |  |  |
| Study selection | 16a | Describe the results of the search and selection process, from the number of records identified in the search to the number of studies included in the review, ideally using a flow diagram. | Results, Study Selection section, p.5 + Figure 1 — 1,748 records identified, 882 duplicates removed, 866 screened, 32 full-text assessed, 22 excluded, 10 studies included; PRISMA flow diagram in Figure 1 |
|  | 16b | Cite studies that might appear to meet the inclusion criteria, but which were excluded, and explain why they were excluded. | Results, Study Selection section, p.5 — 22 full-text exclusions with reasons: no operative outcomes (n=8), single-arm (n=5), no interval LC (n=4), conference abstract (n=2), overlapping cohort (n=2), insufficient data (n=1) |
| Study characteristics | 17 | Cite each included study and present its characteristics. | Results, Study Characteristics section, p.5 + Table 1 — 10 studies (2019–2025): 9 retrospective cohorts (1 PSM), 1 RCT; characteristics including country, sample size, drainage types, severity |
| Risk of bias in studies | 18 | Present assessments of risk of bias for each included study. | Results, p.6 + Supplementary Table S2 — All 9 observational studies rated serious (ROBINS-I) except Masuda 2024 (moderate); Mu 2021 RCT rated some concerns (RoB 2) |
| Results of individual studies | 19 | For all outcomes, present, for each study: (a) summary statistics for each group (where appropriate) and (b) an effect estimate and its precision (e.g. confidence/credible interval), ideally using structured tables or plots. | Results, p.6–7 + Figure 2 + Supplementary Table S5 — Individual study data in forest plots with events, totals, weights, RR/MD with 95% CI for each study |
| Results of syntheses | 20a | For each synthesis, briefly summarise the characteristics and risk of bias among contributing studies. | Results, p.6 + Table 3 — Each outcome includes number of studies, participants, and certainty of evidence incorporating risk of bias |
|  | 20b | Present results of all statistical syntheses conducted. If meta-analysis was done, present for each the summary estimate and its precision (e.g. confidence/credible interval) and measures of statistical heterogeneity. If comparing groups, describe the direction of the effect. | Results, p.6–7 + Table 2 — All pooled estimates with RR/MD, 95% HKSJ CI, P-values, I², Q test results; EUS-GBD vs PTGBD: RR 0.51 [0.23–1.13], I²=0%; EGBS vs PTGBD: RR 1.14 [0.25–5.23], I²=77% |
|  | 20c | Present results of all investigations of possible causes of heterogeneity among study results. | Results, p.6–7 + Supplementary Figure S1 — Subgroup analysis by study design: PSM (Masuda 2024) RR 0.17 vs unadjusted RR 1.78; test for subgroup difference χ²=13.12, P=0.0003, I²=92.4% |
|  | 20d | Present results of all sensitivity analyses conducted to assess the robustness of the synthesized results. | Supplementary Table S5 — HKSJ vs Wald CI, fixed vs random effects, excluding influential studies; results consistent across methods |
| Reporting biases | 21 | Present assessments of risk of bias due to missing results (arising from reporting biases) for each synthesis assessed. | Discussion, Limitations section, p.8 — Publication bias assessment not feasible (<10 studies); GRADE rating includes consideration of publication bias |
| Certainty of evidence | 22 | Present assessments of certainty (or confidence) in the body of evidence for each outcome assessed. | Results, Certainty of Evidence section, p.7 + Table 3 — GRADE Summary of Findings: EUS-GBD conversion LOW, EGBS conversion VERY LOW, EGBS subtotal cholecystectomy VERY LOW |
| **DISCUSSION** |  |  |  |
| Discussion | 23a | Provide a general interpretation of the results in the context of other evidence. | Discussion, p.7–8 — Results integrated with existing drainage efficacy meta-analyses (Boregowda 2023, Mohan 2020, Lyu 2021); EUS-GBD favorable trend consistent with drainage superiority evidence |
|  | 23b | Discuss any limitations of the evidence included in the review. | Discussion, Limitations section, p.8 — Observational design, confounding by indication, small sample sizes, heterogeneous populations, variable definitions |
|  | 23c | Discuss any limitations of the review processes used. | Discussion, Limitations section, p.8 — Publication bias assessment not feasible, limited studies for subgroup analyses, single PSM study |
|  | 23d | Discuss implications of the results for practice, policy, and future research. | Discussion, Clinical Implications section, p.8 + Conclusions, p.9 — When operative outcomes equivalent, prioritize drainage efficacy; EUS-GBD preferred when expertise available; need for larger PSM/RCT studies |
| **OTHER INFORMATION** |  |  |  |
| Registration and protocol | 24a | Provide registration information for the review, including register name and registration number, or state that the review was not registered. | Methods, p.3 + Abstract — PROSPERO: CRD420251232718 |
|  | 24b | Indicate where the review protocol can be accessed, or state that a protocol was not prepared. | Supplementary Document S1 — Protocol available via PROSPERO at https://www.crd.york.ac.uk/prospero/ |
|  | 24c | Describe and explain any amendments to information provided at registration or in the protocol. | Methods, p.3 — "The protocol underwent seven amendments, all documented before examining pooled effect estimates, including changes to population scope, comparison structure, outcome hierarchy, effect measures, and confidence interval methodology." |
| Support | 25 | Describe sources of financial or non-financial support for the review, and the role of the funders or sponsors in the review. | Declarations — No funding received for this study |
| Competing interests | 26 | Declare any competing interests of review authors. | Declarations — All authors declare no competing interests |
| Availability of data, code and other materials | 27 | Report which of the following are publicly available and where they can be found: template data collection forms; data extracted from included studies; data used for all analyses; analytic code; any other materials used in the review. | Declarations — Data extraction forms and analysis data available from corresponding author upon reasonable request; protocol publicly available via PROSPERO |

**Reference:**

Page MJ, McKenzie JE, Bossuyt PM, Boutron I, Hoffmann TC, Mulrow CD, et al. The PRISMA 2020 statement: an updated guideline for reporting systematic reviews. BMJ 2021;372:n71. doi: 10.1136/bmj.n71.

This work is licensed under CC BY 4.0. To view a copy of this license, visit https://creativecommons.org/licenses/by/4.0/
