# Supplementary material for: Operative outcomes of interval cholecystectomy after gallbladder drainage for acute cholecystitis: a systematic review and meta-analysis comparing endoscopic and percutaneous approaches
Source: BMC Surg. 2026 Mar 9;26:268. doi: 10.1186/s12893-026-03644-2 (PMC13085629; doi:10.1186/s12893-026-03644-2)
Supplement: Supplementary file 2 — Supplementary Material 2. [file 12893_2026_3644_MOESM2_ESM.docx]

**Supplementary Materials**

**Supplementary Tables**

**Supplementary Table S1. Complete search strategies for all databases.**

**PubMed/MEDLINE**

| **Line** | **Search terms** |
| --- | --- |
| #1 | "Cholecystitis, Acute"[Mesh] OR "acute cholecystitis"[tiab] OR "calculous cholecystitis"[tiab] |
| #2 | "Cholecystostomy"[Mesh] OR "Drainage"[Mesh:NoExp] OR "gallbladder drainage"[tiab] |
| #3 | "EUS-guided gallbladder drainage"[tiab] OR "EUS-GBD"[tiab] OR "endoscopic ultrasound guided gallbladder drainage"[tiab] |
| #4 | "endoscopic transpapillary gallbladder drainage"[tiab] OR "ETGBD"[tiab] OR "EGBS"[tiab] OR "ENGBD"[tiab] OR "transpapillary gallbladder stent*"[tiab] |
| #5 | "percutaneous cholecystostomy"[tiab] OR "PTGBD"[tiab] OR "percutaneous transhepatic gallbladder drainage"[tiab] |
| #6 | #3 OR #4 OR #5 |
| #7 | "Cholecystectomy, Laparoscopic"[Mesh] OR "laparoscopic cholecystectomy"[tiab] OR "interval cholecystectomy"[tiab] |
| #8 | random*[tiab] OR cohort[tiab] OR "comparative study"[pt] OR propensity[tiab] OR versus[tiab] |
| #9 | #1 AND (#2 OR #6) AND #7 AND #8 |
| #10 | #9 NOT (animals[mh] NOT humans[mh]) |
| **Final** | **#10 --- Results: 528** |

**Embase (Ovid)**

| **Line** | **Search terms** |
| --- | --- |
| 1 | exp acute cholecystitis/ OR acute cholecystitis.ti,ab. |
| 2 | exp cholecystostomy/ OR gallbladder drainage.ti,ab. |
| 3 | EUS-guided gallbladder drainage.ti,ab. OR EUS-GBD.ti,ab. |
| 4 | ETGBD.ti,ab. OR EGBS.ti,ab. OR ENGBD.ti,ab. OR transpapillary gallbladder drainage.ti,ab. |
| 5 | percutaneous cholecystostomy.ti,ab. OR PTGBD.ti,ab. |
| 6 | 3 OR 4 OR 5 |
| 7 | exp laparoscopic cholecystectomy/ OR interval cholecystectomy.ti,ab. |
| 8 | random$.ti,ab. OR cohort.ti,ab. OR comparative study.ti,ab. OR propensity.ti,ab. |
| 9 | 1 AND (2 OR 6) AND 7 AND 8 |
| 10 | 9 NOT (exp animal/ NOT exp human/) |
| 11 | 10 NOT (conference abstract).pt. |
| **Final** | **11 --- Results: 488** |

**Scopus**

| **Component** | **Search terms** |
| --- | --- |
| Population | TITLE-ABS-KEY("acute cholecystitis" OR "calculous cholecystitis") |
| Intervention | TITLE-ABS-KEY("EUS-GBD" OR "ETGBD" OR "EGBS" OR "ENGBD" OR "PTGBD" OR "gallbladder drainage") |
| Outcome | TITLE-ABS-KEY("laparoscopic cholecystectomy" OR "interval cholecystectomy") |
| Study design | TITLE-ABS-KEY(random* OR cohort OR comparative OR propensity OR versus) |
| Limits | PUBYEAR > 1999; DOCTYPE = "ar" OR "re"; NOT animal studies |
| **Final** | **Results: 536** |

**Cochrane CENTRAL**

| **Line** | **Search terms** |
| --- | --- |
| #1 | MeSH descriptor: [Cholecystitis, Acute] explode all trees |
| #2 | ("acute cholecystitis" OR "calculous cholecystitis"):ti,ab,kw |
| #3 | ("EUS-GBD" OR "ETGBD" OR "EGBS" OR "ENGBD" OR "PTGBD" OR "gallbladder drainage"):ti,ab,kw |
| #4 | ("laparoscopic cholecystectomy" OR "interval cholecystectomy"):ti,ab,kw |
| #5 | (#1 OR #2) AND #3 AND #4 |
| **Final** | **#5 with publication date from Jan 2000, in Trials --- Results: 111** |

**ClinicalTrials.gov**

| **Field** | **Search terms** |
| --- | --- |
| Condition | acute cholecystitis OR calculous cholecystitis |
| Other terms | EUS-GBD OR ETGBD OR EGBS OR ENGBD OR PTGBD OR gallbladder drainage |
| Intervention | laparoscopic cholecystectomy OR interval cholecystectomy |
| Study type | Interventional OR Observational |
| First posted | From 01/01/2000 |
| **Final** | **Results: 85** |

**Summary of Search Results**

| **Database** | **Records identified** |
| --- | --- |
| PubMed | 528 |
| Scopus | 536 |
| Embase | 488 |
| Cochrane CENTRAL | 111 |
| ClinicalTrials.gov | 85 |
| **Total** | **1,748** |
| After deduplication | 866 |
| Full-text assessed | 32 |
| Studies included | 10 |

*Search strategies were developed in consultation with a medical librarian and adapted for each database syntax.*

**Supplementary Table S2. Domain-level risk of bias assessment for all included studies.**

**Table S2a. ROBINS-I assessment for observational studies (9 studies)**

| **Study** | **D1 Confounding** | **D2 Selection** | **D3 Classification** | **D4 Deviations** | **D5 Missing data** | **D6 Measurement** | **D7 Reporting** | **Overall** |
| --- | --- | --- | --- | --- | --- | --- | --- | --- |
| Tyberg 2023 | Serious | Moderate | Low | Low | Low | Low | Low | **Serious** |
| Ishii 2023 | Serious | Serious | Low | Low | Low | Low | Low | **Serious** |
| Saumoy 2019 | Serious | Moderate | Low | Low | Low | Low | Low | **Serious** |
| Kaura 2020 | Serious | Moderate | Low | Low | Low | Low | Low | **Serious** |
| Kawano 2021 | Serious | Moderate | Low | Low | Low | Low | Low | **Serious** |
| Kaneta 2022 | Serious | Serious | Low | Low | Low | Low | Low | **Serious** |
| Tanaka 2023 | Serious | Moderate | Low | Low | Low | Low | Low | **Serious** |
| Katsura 2024 | Serious | Moderate | Low | Low | Low | Low | Moderate | **Serious** |
| Masuda 2024 | Moderate | Moderate | Low | Low | Low | Low | Low | **Moderate** |

**Support for judgment (ROBINS-I domains rated above low risk):**

| **Study** | **Domain** | **Judgment** | **Support for judgment** |
| --- | --- | --- | --- |
| Tyberg 2023 | D1 Confounding | Serious | Multicenter retrospective design; no adjustment for confounders (cholecystitis severity, comorbidities, surgeon experience); drainage modality selected based on institutional preference and endoscopist availability |
| Tyberg 2023 | D2 Selection | Moderate | Consecutive patients included but unequal group sizes (46 vs 92); selection into drainage modality potentially influenced by patient factors |
| Ishii 2023 | D1 Confounding | Serious | Single-center retrospective; no adjustment for prognostic factors; EUS-GBD introduced as new technique during study period (temporal confounding) |
| Ishii 2023 | D2 Selection | Serious | Markedly unequal groups (35 EUS-GBD vs 8 PTGBD for laparoscopic-intended cases); very small comparator group limits comparability; PTGBD arm included 11 total patients but only 8 attempted laparoscopically |
| Saumoy 2019 | D1 Confounding | Serious | Single-center retrospective; no adjustment for confounders; drainage modality determined by physician preference and availability |
| Saumoy 2019 | D2 Selection | Moderate | Unequal groups (13 vs 21); consecutive patient enrollment but selection influenced by endoscopic expertise availability |
| Kaura 2020 | D1 Confounding | Serious | Single-center retrospective; no multivariable adjustment; drainage selection influenced by availability of ERCP expertise and patient severity |
| Kaura 2020 | D2 Selection | Moderate | Unequal groups (52 vs 140); consecutive patients from a single institution; EGBS available only when ERCP expertise present |
| Kawano 2021 | D1 Confounding | Serious | Single-center retrospective; no adjustment for confounders; pilot study design with sequential enrollment |
| Kawano 2021 | D2 Selection | Moderate | Small sample (18 vs 10); drainage modality determined by period of treatment and clinical decision |
| Kaneta 2022 | D1 Confounding | Serious | Single-center retrospective; no adjustment for prognostic factors; drainage selection based on clinical judgment |
| Kaneta 2022 | D2 Selection | Serious | Very small intervention group (7 EGBS vs 26 PTGBD); extreme group imbalance suggests systematic selection differences; limited comparability |
| Tanaka 2023 | D1 Confounding | Serious | Single-center retrospective; no multivariable adjustment; drainage modality determined by physician preference |
| Tanaka 2023 | D2 Selection | Moderate | Unequal groups (9 vs 34); consecutive enrollment but small EGBS group reflects evolving institutional practice |
| Katsura 2024 | D1 Confounding | Serious | Single-center retrospective; no adjustment for confounders; drainage selection based on clinical factors and endoscopic availability |
| Katsura 2024 | D2 Selection | Moderate | Unequal groups (14 vs 45); consecutive patients but selection influenced by availability of endoscopic expertise |
| Katsura 2024 | D7 Reporting | Moderate | Excluded planned open cholecystectomy cases from conversion denominator (4 PTGBD, 0 EGBS); asymmetric exclusion may influence conversion rate estimates |
| Masuda 2024 | D1 Confounding | Moderate | Propensity score matching adjusted for measured confounders (age, sex, BMI, comorbidities, cholecystitis severity); however, residual unmeasured confounding possible (e.g., surgeon experience, anatomical factors) |
| Masuda 2024 | D2 Selection | Moderate | PSM produced balanced groups (43 vs 43); however, pre-matching cohort had substantial differences suggesting confounding by indication; post-matching balance adequate for measured variables |

**Table S2b. RoB 2 assessment for randomized controlled trial (Mu 2021)**

| **Domain** | **Judgment** | **Support for judgment** |
| --- | --- | --- |
| D1. Randomization process | Some concerns | Computer-generated randomization list with sealed opaque envelopes (adequate allocation concealment); however, small sample (n=30 randomized, n=22 surgical cohort) with potential for baseline imbalance despite randomization |
| D2. Deviations from intended interventions | Some concerns | Open-label trial: patients, treating physicians, and endoscopists aware of allocation; only outcome assessors were masked. Knowledge of allocation may have influenced co-interventions and decisions regarding surgical approach |
| D3. Missing outcome data | Low | All 22 patients in the surgical cohort completed follow-up and were readmitted for interval cholecystectomy; complete outcome data available |
| D4. Measurement of the outcome | Low | Outcome assessors masked to allocation; operative outcomes (conversion, operative time, blood loss) are objective measures with low risk of ascertainment bias |
| D5. Selection of the reported result | Some concerns | Primary outcome was abdominal pain score (NRS), not operative outcomes; cholecystectomy outcomes were secondary/exploratory endpoints reported without pre-specified analysis plan for surgical outcomes; multiple operative outcomes reported without correction for multiplicity |
| **Overall** | **Some concerns** | **Driven by open-label design, small sample size, and reporting of surgical outcomes as secondary endpoints without pre-specified analysis plan** |

*Abbreviations: D1–D7 for ROBINS-I: D1, bias due to confounding; D2, bias in selection of participants into the study; D3, bias in classification of interventions; D4, bias due to deviations from intended interventions; D5, bias due to missing data; D6, bias in measurement of outcomes; D7, bias in selection of the reported result. D1–D5 for RoB 2: D1, bias arising from the randomization process; D2, bias due to deviations from intended interventions; D3, bias due to missing outcome data; D4, bias in measurement of the outcome; D5, bias in selection of the reported result. NRS, numeric rating scale; PSM, propensity score-matched; RoB 2, Cochrane Risk of Bias tool version 2; ROBINS-I, Risk Of Bias In Non-randomised Studies of Interventions.*

**Supplementary Table S3. Data extraction audit trail with source citations.**

Page numbers and table references for each extracted data point to ensure reproducibility.

| **Study** | **Conversion Source** | **Subtotal CCY Source** | **Operative Time Source** | **Blood Loss Source** |
| --- | --- | --- | --- | --- |
| Tyberg 2023 | Table 2, p.5 | NR | Table 2, p.4 | NR |
| Ishii 2023 | Table 2, p.5 | NR | Table 2, p.5 | Table 2, p.5 |
| Saumoy 2019 | Table 2, p.4 | NR | Table 3, p.4 | Table 3, p.4 |
| Kaura 2020 | Table 3, p.999 | Results, p.5 | NR | Table 3, p.999 |
| Kawano 2021 | Table 2, p.4 | NR | Table 3, p.5 | Table 3, p.5 |
| Kaneta 2022 | Table 4, p.5 | Table 4, p.5 | Table 4, p.4 | Table 4, p.4 |
| Tanaka 2023 | Table 4, p.7 | Table 2, p.5 | Table 2, p.5 | Table 2, p.5 |
| Katsura 2024 | Table 3 | Table, p.2 | Table, p.1 | Table, p.1 |
| Masuda 2024 | Table 3 | Table 4, p.5 | Table 4, p.6 | Table 4, p.6 |
| Mu 2021 | Table 5, p.5 | NR | Table 5, p.5 | Table 5, p.5 |

*Abbreviations: CCY, cholecystectomy; NR, not reported.*

**Supplementary Table S4. Median-to-mean conversion calculations.**

Transformation of continuous outcomes using validated formulas for studies reporting median with range or interquartile range.

**Conversion Methods:**

- *Wan C1 (for median with range, n ≤ 25):* Mean ≈ (a + 2m + b) / 4; SD ≈ (b − a) / (2 × Φ⁻¹[(n − 0.375)/(n + 0.25)]), where a = minimum, m = median, b = maximum
- *Luo (for median with IQR):* Mean ≈ (q₁ + m + q₃) / 3; SD ≈ (q₃ − q₁) / (2 × Φ⁻¹[(0.75n − 0.125)/(n + 0.25)]), where q₁, q₃ = quartiles

| **Study** | **Outcome** | **n** | **Original Format** | **Method** | **Converted Mean** | **Converted SD** |
| --- | --- | --- | --- | --- | --- | --- |
| Kawano 2021 | Operative time (EGBS) | 18 | 209 (107--357) | Wan C1 | 211.3 | 62.5 |
| Kawano 2021 | Operative time (PTGBD) | 10 | 161 (130--273) | Wan C1 | 168.0 | 35.8 |
| Kaneta 2022 | Operative time (EGBS) | 7 | 166 (76--299) | Wan C1 | 171.3 | 55.8 |
| Kaneta 2022 | Operative time (PTGBD) | 26 | 143 (75--264) | Wan C1 | 147.8 | 47.3 |
| Tanaka 2023 | Operative time (EGBS) | 9 | 175 (141--262) | Wan C1 | 178.7 | 30.3 |
| Tanaka 2023 | Operative time (PTGBD) | 34 | 147 (65--359) | Wan C1 | 168.1 | 73.5 |
| Katsura 2024 | Operative time (EGBS) | 14 | 160 [141--205] | Luo | 168.7 | 47.4 |
| Katsura 2024 | Operative time (PTGBD) | 45 | 137 [113--164] | Luo | 138.0 | 37.8 |
| Mu 2021 | Operative time (ENGBD) | 11 | 50 [47--90] | Luo | 62.3 | 31.9 |
| Mu 2021 | Operative time (PTGBD) | 11 | 70 [50--104] | Luo | 74.7 | 40.0 |
| Mu 2021 | Blood loss (ENGBD) | 11 | 15 [5--20] | Luo | 13.3 | 11.1 |
| Mu 2021 | Blood loss (PTGBD) | 11 | 40 [20--70] | Luo | 43.3 | 37.0 |

*Note: Similar conversions were applied for blood loss data. Complete calculation spreadsheet available from corresponding author.*

*References: Wan C, et al. BMC Med Res Methodol. 2014;14:135. Luo D, et al. Stat Methods Med Res. 2018;27(6):1785-1805.*

**Supplementary Table S5. Sensitivity analyses results.**

Comparison of primary HKSJ analysis with Wald-type confidence intervals, fixed-effects models, and exclusion of influential studies.

| **Comparison** | **Outcome** | **Primary (HKSJ)** | **Sensitivity (Wald)** | **Fixed Effects** | **Excluding Influential Study** |
| --- | --- | --- | --- | --- | --- |
| EUS-GBD vs PTGBD | Conversion | RR 0.51 [0.23--1.13], I²=0% | RR 0.51 [0.22--1.17] | RR 0.52 [0.22--1.20] | RR 0.32 [0.00--58.73]ᵃ |
| EUS-GBD vs PTGBD | Operative time | MD −59.4 [−159.5, 40.6], I²=82% | MD −59.4 [−102.4, −16.5] | MD −72.3 [−86.3, −58.3] | MD −45.5 [−499.6, 408.6]ᵃ |
| EUS-GBD vs PTGBD | Blood loss | MD −57.8 [−309.5, 193.9], I²=0% | MD −57.8 [−104.0, −11.6] | MD −57.8 [−104.0, −11.6] | --- |
| EGBS vs PTGBD | Conversion | RR 1.14 [0.25--5.23], I²=77% | RR 1.14 [0.40--3.24] | RR 1.04 [0.70--1.54] | RR 1.86 [0.74--4.69]ᵇ |
| EGBS vs PTGBD | Subtotal CCY | RR 1.16 [0.61--2.18], I²=0% | RR 1.16 [0.73--1.84] | RR 1.19 [0.74--1.91] | RR 1.80 [0.32--10.17]ᶜ |
| EGBS vs PTGBD | Operative time | MD +7.6 [−43.3, 58.4], I²=85% | MD +7.6 [−35.0, 50.1] | MD −1.6 [−17.2, 14.0] | MD +26.8 [7.7, 45.8]ᵈ |
| EGBS vs PTGBD | Blood loss | MD −9.4 [−43.9, 25.1], I²=75% | MD −9.4 [−37.1, 18.3] | MD −18.5 [−30.7, −6.2] | MD −17.2 [−29.8, −4.6]ᵈ |
| EGBS vs PTGBD | Major complications | RR 1.02 [0.14--7.31], I²=15% | RR 1.02 [0.44--2.39] | RR 1.02 [0.50--2.07] | RR 1.02 [0.50--2.07]ᵉ |

ᵃExcluding Tyberg 2023 (anomalous SD=7.7 min, CV=9%); k=2 with df=1 results in extremely wide HKSJ CI. ᵇExcluding Masuda 2024 (only PSM study); I² reduced from 77% to 3%, confirming Masuda as source of heterogeneity; T=2.32, df=3, P=0.10. ᶜExcluding Katsura 2024 (dominant weight 79.1%). ᵈExcluding Masuda 2024 (only study with original mean±SD); fixed-effect model used, I²=0% after exclusion. ᵉExcluding double-zero studies (Kawano 2021, Kaneta 2022).

*Note: HKSJ confidence intervals are appropriately wider than Wald-type, reflecting genuine uncertainty with small numbers of studies (k=2--6). The dramatic I² reduction when excluding Masuda 2024 (77%→3%) demonstrates that observed heterogeneity reflects confounding by indication rather than true clinical variability. Forest plots for all sensitivity analyses are provided in the accompanying Supplementary Table S5 document.*

**Supplementary Figures**

**Supplementary Figure S1. Subgroup analysis by study design for EGBS versus PTGBD conversion to open cholecystectomy.**


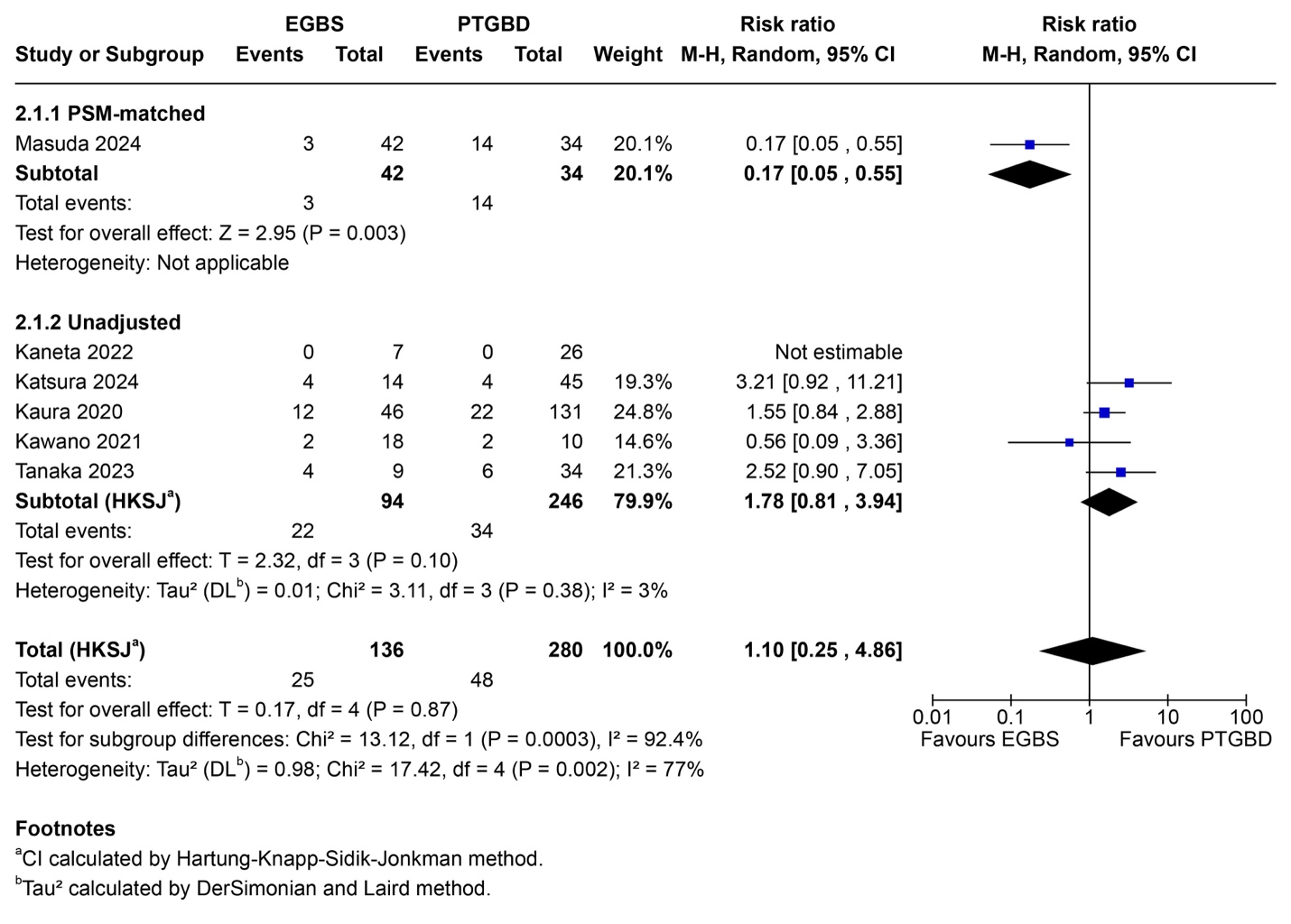


Forest plot showing subgroup analysis stratified by study design (propensity score-matched vs unadjusted retrospective cohorts). The PSM-matched subgroup (Masuda 2024 only; RR 0.17, 95% CI 0.05--0.55; P=0.003) shows results favoring EGBS, while the unadjusted subgroup (k=5; RR 1.78, 95% HKSJ CI 0.81--3.94; I²=3%; P=0.10) shows the opposite trend. Test for subgroup difference: χ²=13.12, df=1, P=0.0003, I²=92.4%. This striking discordance confirms that the observed heterogeneity reflects confounding by indication rather than true clinical variability.

**Supplementary Figure S2. Sensitivity analysis: EUS-GBD versus PTGBD operative time excluding Tyberg 2023.**


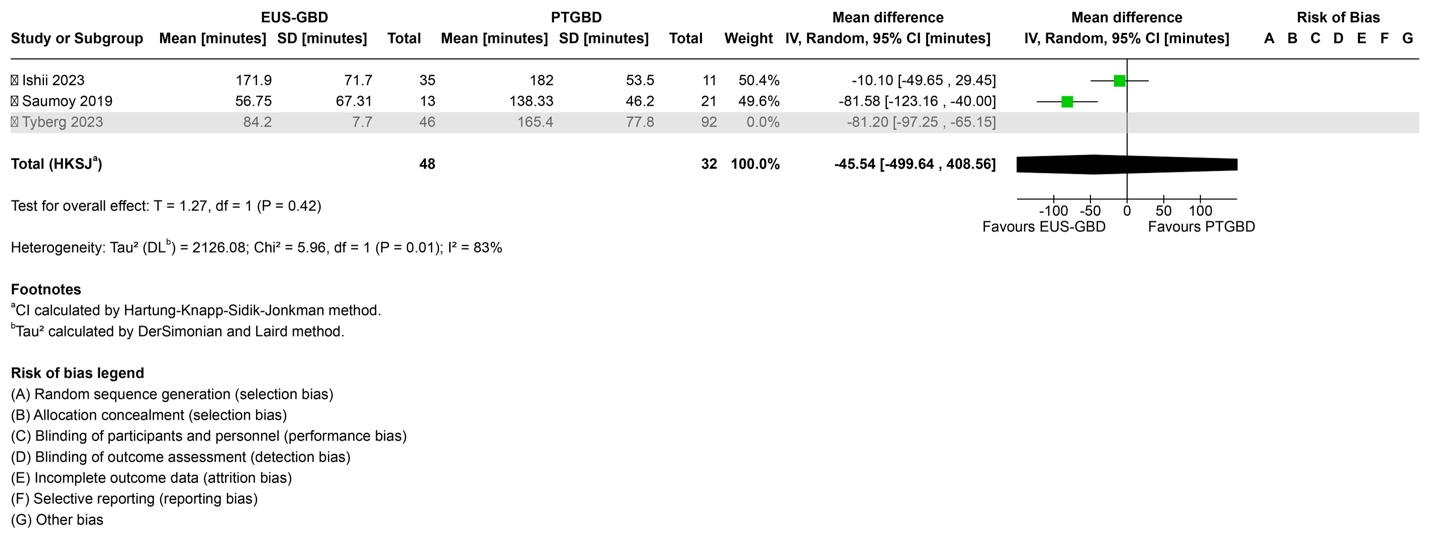


Forest plot excluding Tyberg 2023 due to anomalously low standard deviation (SD=7.7 minutes for a mean of 84.2 minutes, coefficient of variation 9%). Remaining studies (Ishii 2023: MD −10.1 min; Saumoy 2019: MD −81.6 min) show pooled MD −45.5 min (95% HKSJ CI −499.6 to 408.6; I²=83%). The extremely wide HKSJ confidence interval reflects genuine uncertainty with only k=2 studies (df=1), demonstrating that exclusion of the dominant study eliminates precision while the direction of effect remains favorable toward EUS-GBD.

**Supplementary Documents**

**Supplementary Document S1. PRISMA 2020 checklist.**

Completed checklist demonstrating compliance with the Preferred Reporting Items for Systematic Reviews and Meta-Analyses guidelines.

*Note: The study protocol with complete amendment history is publicly available through PROSPERO (CRD420251232718) at https://www.crd.york.ac.uk/prospero/.*
